# Supplementary material for: One out of four patients with pancreatic cancer experience psychological symptoms: A systematic review and meta-analysis
Source: PLoS One. 2026 May 27;21(5):e0348435. doi: 10.1371/journal.pone.0348435 (PMC13215498; doi:10.1371/journal.pone.0348435)
Supplement: S1 Documentum — (PDF) [file pone.0348435.s001.pdf]

Search key

The following search key was used to identify eligible articles: (pancreatic cancer) AND (psychology OR psych* OR mental OR depression OR depress* OR anxiety OR anxi* OR distress OR stress OR stress* OR sleep OR sleep* OR panic OR fear OR emotional OR hope OR hope* OR suicide OR despair).
