# Supplementary material for: One out of four patients with pancreatic cancer experience psychological symptoms: A systematic review and meta-analysis
Source: PLoS One. 2026 May 27;21(5):e0348435. doi: 10.1371/journal.pone.0348435 (PMC13215498; doi:10.1371/journal.pone.0348435)
Supplement: S2 Documentum — (PDF) [file pone.0348435.s002.pdf]

Data synthesis – detailed methods

We used a random intercept logistic regression model (aka. glmm, with the logit transformation of the observed proportions) for meta-analysis to pool proportions. To estimate the heterogeneity variance measure τ^2, a maximum likelihood method was used. We used a Hartung-Knapp adjustment[1] for CIs (if it is more conservative than the classical one, as recommended by Jackson et el.[2] as hybrid method 2) for each estimate. Prediction interval determination for proportion outcomes was based on t-distribution.
The Agresti-Coull[3] method was used for confidence interval calculation of proportion for individual studies and shown on forest plots.
Outlier and influence analyses were carried out following the recommendations of Harrer et al.[4], using leave-one-out analyses (Figure S3.-8.) For assessing the small study publication bias, funnel plots were used with visual inspection. (Figure S1-2.) Additionally, we performed Egger's test (at a significance level of 10% as a small study number) using the Peters method[5], but it was critically handled if the study number was below ten or the study effects showed high heterogeneity.

REFERENCES

1 IntHout, J., Ioannidis, J. P. & Borm, G. F. The Hartung-Knapp-Sidik-Jonkman method for random effects meta-analysis is straightforward and considerably outperforms the standard DerSimonian-Laird method. *BMC Med Res Methodol* **14**, 25, doi:10.1186/1471-2288-14-25 (2014).

2 Jackson, D., Law, M., Rücker, G. & Schwarzer, G. The Hartung-Knapp modification for random-effects meta-analysis: A useful refinement but are there any residual concerns? *Stat Med* **36**, 3923-3934, doi:10.1002/sim.7411 (2017).

3 Agresti, A. & Coull, B. A. Approximate is Better than "Exact" for Interval Estimation of Binomial Proportions. *The American Statistician* **52**, 119-126, doi:10.1080/00031305.1998.10480550 (1998).

4 Harrer, M., Cuijpers, P., Furukawa, T. & Ebert, D. *Doing Meta-Analysis with R: A Hands-On Guide*. (2021).

5 Peters, J. L., Sutton, A. J., Jones, D. R., Abrams, K. R. & Rushton, L. Comparison of two methods to detect publication bias in meta-analysis. *Jama* **295**, 676-680, doi:10.1001/jama.295.6.676 (2006).
