# Supplementary material for: One out of four patients with pancreatic cancer experience psychological symptoms: A systematic review and meta-analysis
Source: PLoS One. 2026 May 27;21(5):e0348435. doi: 10.1371/journal.pone.0348435 (PMC13215498; doi:10.1371/journal.pone.0348435)
Supplement: S3 Documentum — This table contains the complete dataset used to generate all results presented in the manuscript. ‘Short’-within six months of cancer diagnosis; ‘long’-beyond six months from cancer diagnosis. ROB- Risk of bias. (PDF) [file pone.0348435.s003.pdf]

Outcome	Study	Include	Patients with symptoms	Total number of patients	Subgroup	ROB	Time category
Depression	Akizuki, et al., 2016	yes	3	110	total	low	short
Anxiety	Akizuki, et al., 2016	yes	2	110	total	low	short
Anxiety	Batra, et al. , 2021	yes	29	94	total	moderate	long
Anxiety	Boyd, et al. , 2012	yes	21	22	total	moderate	long
Anxiety	Brinzenthofe , et al., 2009	yes	17	185	total	low	NA
Anxiety	Clark, et al., 2010	yes	89	304	total	low	short
Anxiety	Del Piccolo, et al. , 2021	yes	180	400	total	moderate	short
Anxiety	Fras, et al. , 1967	yes	16	35	total	high	short
Anxiety	Harris, et al., 2021	yes	1167	10378	total	moderate	long
Anxiety	Jacobbson, et al. , 1971	no	4	57	total	NA	NA
Anxiety	Janda, et al. , 2017	yes	20	136	total	low	long
Anxiety	Lelond, et al. , 2021	yes	62	123	total	moderate	short
Anxiety	Mehnert, et al. , 2014	yes	3	52	total	low	short
Anxiety	Seoud, et al. , 2020	yes	4740	10220	total	low	long
Anxiety	Carruba, et al. ,2022	yes	4	11	moderate	low	NA
Anxiety	Carruba, et al. ,2022	yes	5	11	severe	low	NA
Anxiety	Carruba, et al. ,2022	yes	9	11	total	low	NA
Anxiety	Cui, et al. ,2023	yes	132	209	total	low	NA
Anxiety	Hussain, et al. ,2023	no	49,46%	NA	NA	moderate	short
Anxiety	Salm, et al. ,2021	yes	6	80	total	low	long
Anxiety	Subramaniam, et al. ,2024	yes	1068	4029	total	low	short
Anxiety	Vehling, et al. ,2022	yes	4	50	total	low	long
Anxiety	Zhang, et al. ,2022	yes	36	100	mild	low	NA
Anxiety	Zhang, et al. ,2022	yes	12	100	moderate	low	NA
Anxiety	Zhang, et al. ,2022	yes	5	100	severe	low	NA
Anxiety	Zhang, et al. ,2022	yes	53	100	total	low	NA
Depression	Batra, et al., 2021	yes	27	94	total	moderate	long
Depression	Boyd, et al., 2012	yes	7	22	mild	moderate	long
Depression	Boyd, et al., 2012	yes	5	22	moderate	moderate	long
Depression	Boyd, et al., 2012	yes	1	22	severe	moderate	long

Outcome	Study	Include	Patients with symptoms	Total number of patients	Subgroup	ROB	Time category
Depression	Akizuki, et al., 2016	yes	3	110	total	low	short
Depression	Boyd, et al., 2012	yes	13	22	total	moderate	long
Depression	Brintzenhofe, et al., 2009	yes	18	185	total	low	NA
Depression	Clark, et al., 2010	yes	87	304	total	low	short
Depression	Del Piccolo, et al., 2021	yes	180	400	total	moderate	short
Depression	Fras, et al., 1967	yes	23	50	total	high	short
Depression	Godby, et al. , 2020	yes	14	88	total	low	short
Depression	Harris, et al., 2021	yes	1581	10378	total	moderate	long
Depression	Hartung, et al. , 2017	yes	28	82	total	low	short
Depression	Hartung, et al. , 2017	yes	25	82	mild	low	short
Depression	Hartung, et al. , 2017	yes	17	82	severe	low	short
Depression	Hartung, et al. , 2017	yes	40	82	moderate	low	short
Depression	Jacobsson, et al. , 1971	no	10	57	total	NA	NA
Depression	Janda, et al., 2017	yes	22	136	mild	low	long
Depression	Janda, et al., 2017	yes	20	136	severe	low	long
Depression	Janda, et al., 2017	yes	42	136	total	low	long
Depression	Kim, et al., 2023	yes	13	36	mild	low	short
Depression	Kim, et al., 2023	yes	6	36	moderate	low	short
Depression	Kim, et al., 2023	yes	6	36	severe	low	short
Depression	Kim, et al., 2023	yes	25	36	total	low	short
Depression	Pezzili, et al., 2017	yes	5	22	mild	moderate	short
Depression	Pezzili, et al., 2017	yes	2	22	moderate	moderate	short
Depression	Pezzili, et al., 2017	yes	1	22	severe	moderate	short
Depression	Pezzili, et al., 2017	yes	8	22	total	moderate	short
Depression	Seoud, et al. , 2020	yes	8130	62450	total	low	long
Depression	Carruba, et al. ,2022	yes	6	11	moderate	low	NA
Depression	Carruba, et al. ,2022	yes	4	11	severe	low	NA
Depression	Carruba, et al. ,2022	yes	10	11	total	low	NA
Depression	Hussain, et al. ,2023	no	55,97%	NA	NA	moderate	short

Outcome	Study	Include	Patients with symptoms	Total number of patients	Subgroup	ROB	Time category
Depression	Akizuki, et al., 2016	yes	3	110	total	low	short
Depression	Salm, et al. ,2021	yes	16	80	total	low	long
Depression	Subramaniam, et al. ,2024	yes	1034	4029	total	low	short
Depression	Vehling, et al. ,2022	yes	14	50	total	low	long
Depression	Yeo, et al. ,2023	yes	49	403	total	low	NA
Distress	Carlson, et al., 2004	yes	41	112	total	low	short
Distress	Carlson, et al., 2019	yes	89	148	total	low	short
Distress	Clark, et al., 2010	yes	80	304	total	low	short
Distress	Dai, et al., 2019	yes	572	2043	total	low	short
Distress	Hohmann, et al. ,2022	yes	4	11	total	low	NA
Distress	Yeo, et al. ,2023	yes	93	128	total	low	NA
Fatigue	Clark, et al., 2010	yes	97	304	total	low	short
Fatigue	Fras, et al., 1967	yes	5	46	total	high	short
Fatigue	Lelond, et al., 2021	yes	70	123	total	moderate	short
Fatigue	Seoud, et al., 2020	yes	4950	10220	total	low	long
Fatigue	Cui, et al. ,2023	yes	154	209	total	low	NA
Fatigue	Schmidt, et al. ,2020	yes	18	33	total	low	NA
Fatigue	Yeo, et al. ,2023	yes	77	403	total	low	NA
Sleep disturbances	Boyd, et al., 2012	yes	11	22	total	moderate	long
Sleep disturbances	Jacobbson, et al., 1971	no	7	57	total	NA	NA
Sleep disturbances	Seoud, et al., 2020	yes	4230	10220	total	low	long
Sleep disturbances	Yeo, et al. ,2023	yes	55	403	total	low	NA
