# Supplementary material for: One out of four patients with pancreatic cancer experience psychological symptoms: A systematic review and meta-analysis
Source: PLoS One. 2026 May 27;21(5):e0348435. doi: 10.1371/journal.pone.0348435 (PMC13215498; doi:10.1371/journal.pone.0348435)
Supplement: S4 Documentum — (PDF) [file pone.0348435.s004.pdf]

Author/Year	Study Title	Reason of exclusion
**Abdel-Rahman (2019)**	Socioeconomic predictors of suicide risk among cancer patients in the United States: A population-based study	No data for prevalence
**Akbaş & Yilmaz Eker (2025)**	The impact of malignancy on death anxiety and psychological well-being in middle-aged and older patients undergoing abdominal surgery: a quasi-experimental study	No data for PC
**Akpoviroro et al. (2022)**	Factors influencing no treatment decisions in advanced stage cancers	No data for prevalence
**Akui et al. (2022)**	Associations between insomnia and central sensitization in cancer survivors undergoing opioid therapy for chronic cancer pain: A STROBE-compliant prospective cohort study	No data for PC
**Al-Obaidi et al. (2022)**	Pain among older adults with gastrointestinal malignancies- results from the cancer and aging resilience evaluation (CARE) Registry	No data for PC
**Alacacioğlu et al. (2007)**	Changes in anxiety levels patients with cancer receiving chemotherapy	No data for PC
**Alacacioglu et al. (2013)**	Depression and anxiety in cancer patients and their relatives	No data for PC
**Alsirafy et al. (2022)**	Not Telling Patients Their Cancer Diagnosis in Egypt: Is It Associated With Less Anxiety and Depression and Better Quality of Life?	No data for PC
**Applebaum et al. (2014)**	Optimism, social support, and mental health outcomes in patients with advanced cancer	No data for PC
**Aslakson et al. (2023)**	Effect of Perioperative Palliative Care on Health-Related Quality of Life Among Patients Undergoing Surgery for Cancer	Ineligible study design
**Awunti et al. (2023)**	Evaluation of psychotropic medication usage patterns in patients with pancreatic cancer by race/ethnicity	Ineligible study design

**Bahçecioğlu Turan et al. (2023)**	The Effects of Self-Acupressure on Pain, Fatigue, and Sleep Quality in Colon and Pancreatic Cancer Patients Receiving Chemotherapy: A Randomized Controlled Study	Ineligible study design
**Bar-Sela et al. (2015)**	The effect of complementary and alternative medicine on quality of life, depression, anxiety, and fatigue levels among cancer patients during active oncology treatment: phase II study	No data for PC
**Bassaly et al. (2024)**	Symptom burden and management in patients with end stage liver disease versus pancreatic cancer: A comparative analysis	No data for prevalence
**Batty et al. (2017)**	Psychological distress in relation to site specific cancer mortality: Pooling of unpublished data from 16 prospective cohort studies	No data for prevalence
**Ben Kridis et al. (2023)**	Evaluation of fatigue in Eldery patients	Ineligible study design
**Benny et al. (2022)**	Population-based comparison of cancer survival outcomes in patients with and without psychiatric disorders	No data for prevalence
**Breitbart et al. (2015)**	Meaning-centered group psychotherapy: An effective intervention for improving psychological well-being in patients with advanced cancer	No data for PC
**Breitbart et al. (2018)**	Individual meaning-centered psychotherapy for the treatment of psychological and existential distress: A randomized controlled trial in patients with advanced cancer	No data for PC
**Bubis et al. (2020)**	Patient-Reported Symptom Severity Among 22,650 Cancer Outpatients in the Last Six Months of Life	No data for PC
**Buck et al. (2020)**	Examining the Relationship between Patient Fatigue-Related Symptom Clusters and Carer Depressive Symptoms in Advanced Cancer Dyads: A Secondary Analysis of a Large Hospice Data Set	No data for prevalence

**Calderón et al. (2019):**	Quality of life, coping, and psychological and physical symptoms after surgery for non-metastatic digestive tract cancer	No data for PC
**Chen & Maitra (2023)**	Anxiolytics cause anxiety in pancreatic cancer	No data for prevalence
**Chida et al. (2024)**	The Effectiveness and Feasibility of Palliative Care via Video Telemedicine for Patients with Advanced Cancer…	No data for PC
**Cloyd et al. (2024)**	Early Palliative Care Following Aborted Cancer Surgery: Results of a Prospective Feasibility Trial	Ineligible study design
**Cohen et al. (2022)**	Survivorship care needs in upper gastrointestinal patients following curative surgery and adjuvant treatment	No data for PC
**Conversano et al. (2020)**	Retrospective analyses of psychological distress and defense style among cancer patients	No data for PC
**Cornetta et al. (2023)**	Telehospice for Cancer Patients Discharged from a Tertiary Care Hospital in Western Kenya	No data for PC
**Dallı et al. (2023)**	The effect of two different types of music played to cancer patients during chemotherapy on anxiety, nausea, and satisfaction levels	No data for PC
**Dengsø et al. (2020)**	Increased psychological symptom burden in patients with pancreatic cancer: A population-based cohort study	Ineligible study design
**Dengsø et al. (2023)**	Physical and psychological symptom burden in patients and caregivers during follow-up care after curative surgery for cancers in the pancreas, bile ducts or duodenum	No data for prevalence
**Doherty et al. (2019)**	Exploring the role of psychosocial care in value-based oncology: Results from a survey of 3000 cancer patients and survivors	No data for PC
**Endeshaw et al. (2022)**	Depression, anxiety and their associated factors among patients with cancer receiving treatment at oncology	No data for PC

	units in Amhara Region, Ethiopia: a cross-sectional study	
**Ferrell et al. (2021)**	A Palliative Care Intervention for Patients on Phase 1 Studies	No data for PC
**Galouzis et al. (2025)**	Quality of life and social health in patients after pancreatic surgery	No data for prevalence
**Gehrels et al. (2023)**	Longitudinal health-related quality of life in patients with pancreatic cancer stratified by treatment: a nationwide cohort study	No data for prevalence
**Eggers et al. (2023)**	Exploring Pain, Quality of Life, and Emotional Well-Being in Patients with Advanced Pancreatic Cancer Practicing Spiritual Meditation: A Pilot Study	No data for prevalence
**Gerritsen et al. (2015)**	Developing a core set of patient-reported outcomes in pancreatic cancer: A Delphi survey	No data for prevalence
**Giglio et al. (2022)**	Effects of an Intrathecal Drug Delivery System Connected to a Subcutaneous Port on Pain, Mood and Quality of Life in End Stage Cancer Patients: An Observational Study	No data for PC
**Goess et al. (2024)**	Correlation of intratumoral mast cell quantity with psychosocial distress in patients with pancreatic cancer - the PancStress study	Ineligible study design
**Goldberg et al. (2023)**	Exploring the Relationship Between Health-Illness Transition Experiences and Distress Among Patients With Pancreatic Cancer	No data for prevalence
**Hagan et al. (2017)**	Coping in Patients With Incurable Lung and Gastrointestinal Cancers: A Validation Study of the Brief COPE	No data for PC
**Harms et al. (2023)**	Anxiety in patients with gastrointestinal cancer undergoing primary surgery	No data for PC
**Hau et al. (2023)**	Trajectories of immune-related serum proteins and quality of life in patients with pancreatic and other periampullary cancer: the CHAMP study	No data for PC

**Hossain et al. (2023)**	Age-related differences in symptom distress among patients with cancer	No data for PC
**Hsu et al. (2024)**	Changes in nutritional status and fatigue and their associations with quality of life in patients with pancreatic cancer after surgery: A 12-month longitudinal study	Ineligible study design
**Ibrahim et al. (2024):**	Characterizing the Physical and Psychological Experiences of Newly Diagnosed Pancreatic Cancer Patients	No data for prevalence
**Jarvis et al. (2021)**	Comorbid depression in surgical cancer patients associated with non-routine discharge and readmission	No data for PC
**Kang et al. (2022)**	Prevalence of Psychological Symptoms in Patients Undergoing Pancreatoduodenectomy and Results of a Distress Management System: A Clinic-Based Study	No data for PC
**Katayama et al. (2023)**	Suicidal Ideation Among Patients with Gastrointestinal Cancer	No data for PC
**Kim et al. (2023)**	Effects of cancer stigma on quality of life of patients with hepatobiliary and pancreatic cancer	No data for prevalence
**Kinslow et al. (2024)**	Prognosis and risk of suicide after cancer diagnosis	No data for prevalence
**Kleisiaris et al. (2023)**	Psychological Distress and Concerns of In-Home Older People Living with Cancer and Their Impact on Supportive Care Needs: An Observational Survey	No data for PC
**Kummer et al. (2022)**	Qualitative study in pancreatic cancer: Patient-reported symptoms, disease impact, and HRQoL	Ineligible study design
**Laryionava et al. (2019)**	Cancer Patients' Preferences for either Quality of Life or a Longer Life Determine Their Willingness to Talk about Forgoing Cancer-Specific Treatment	No data for PC
**Li et al. (2023)**	Effect of intimacy and dyadic coping on psychological distress in pancreatic cancer patients and spousal caregivers	No data for prevalence

**Liu & Chen (2024):**	Predictors of Psychiatric Complications in Patients with Pancreatic Cancer: A Retrospective Cohort Study	No data for prevalence
**Ma et al. (2022)**	Effect of Multidisciplinary Team Collaborative Nursing Model Combined with Mind Mapping Teaching Method on Postoperative Complications and Mental Health of Patients with Advanced Pancreatic Cancer	No data for prevalence
**Marinelli et al. (2023)**	Preoperative Anxiety in Patients with Pancreatic Cancer: What Contributes to Anxiety Levels in Patients Waiting for Surgical Intervention	No data for prevalence
**Mi et al. (2024)**	A Randomized Controlled Trial of Mindfulness Meditation Combined With BrainLink Intelligent Biofeedback Instrument on Pancreatic Cancer Patients Under Chemotherapy	No data for prevalence
**Miao (2022)**	The Effect of Comfort Care on Postoperative Quality of Life, Psychological Status, and Satisfaction of Pancreatic Cancer Patients	No data for prevalence
**Miccinesi et al. (2023)**	Assessing suffering of patients on cancer treatment and of those no longer treated using ESAS–Total Care (TC)	No data for PC
**Michalek et al. (2023)**	Suicide after a Diagnosis of Cancer: Follow-Up of 1.4 Million Individuals, 2009–2019	No data for PC
**Mooney et al. (2022)**	A randomized control trial to determine necessary intervention elements to achieve optimal symptom outcomes for a remote symptom management system	No data for prevalence
**Nagpal et al. (2024)**	SMART-ESAS: Smartphone Monitoring and Assessment in Real Time of Edmonton Symptom Assessment System Scores for Patients With Cancer	No data for PC
**Neuzillet et al. (2023)**	Effect of Adapted Physical Activity in Patients With Advanced Pancreatic Cancer: The APACaP GERCOR Randomized Trial	No data for prevalence
**Park et al. (2024)**	Effects of an urban forest healing program on cancer-related fatigue in cancer survivors	No data for PC
**Petit et al. (2024)**	PCR24 Exploring the Content and Psychometric Validity of Clinical Outcome Assessments in Pancreatic Ductal	No data for prevalence

	Adenocarcinoma Versus the Patient Reported Symptoms and Impacts	
**Pichardo et al. (2024)**	Patient-reported and clinical outcomes among patients with pancreatic cancer	No data for prevalence
**Rodríguez-González et al. (2023)**	Mental Adjustment, Functional Status, and Depression in Advanced Cancer Patients	No data for PC
**Schweig et al. (2024)**	Effects of a Mindfulness-Based Intervention on Event-Related Potentials (P3) and Depressive Symptoms in Oncological Patients	No data for PC
**Shannon et al. (2024)**	Quality of Life and Real-time Patient Experience During Neoadjuvant Therapy: A Prospective Cohort Study	No data for PC
**Sharma et al. (2024)**	Prevalence of Depression and Anxiety and their Associated Factors in Patients Attending Palliative Care Centre	No data for PC
**Shin et al. (2021)**	Physical and psychological symptoms and signs in dying digestive tract cancer patients: the East Asian collaborative cross-cultural Study to Elucidate the Dying process (EASED)	No data for PC
**Shrestha et al. (2024)**	Comprehensive assessment of pain characteristics, quality of life, and pain management in cancer patients: a multi-center cross-sectional study	No data for prevalence
**Sitte et al. (2024)**	Correlation of intratumoral mast cell quantity with psychosocial distress in patients with pancreatic cancer: the PancStress study	Ineligible study design
**Stevens et al. (2022)**	Characterizing the patient experience during neoadjuvant therapy for pancreatic ductal adenocarcinoma: A qualitative study	Ineligible study design
**Tang et al. (2016)**	Prevalence of severe depressive symptoms increases as death approaches and is associated with disease burden, tangible social support, and high self-perceived burden to others	No data for PC

**Tayefeh et al. (2021)**	Mood status in patients with gastrointestinal tract cancer undergoing radiotherapy: A randomized cross-sectional study	No data for PC
**Ten Winkel et al. (2024)**	Patient-reported outcomes at three months after pancreatic surgery for benign and malignant diseases - A prospective observational study	No data for prevalence
**Thai et al. (2024)**	Geriatric Assessment Impairment Profiles and Mortality in Older Adults With Gastrointestinal Cancers: Latent Class Analysis of the CARE Registry	No data for PC
**Velasco-Durántez et al. (2023)**	The Relationship between Therapeutic Alliance and Quality of Care in Patients with Advanced Cancer in Spain	No data for PC
**Ward et al. (2022)**	Impact of Neoadjuvant Treatment on Psychosocial Wellbeing in Patients with Pancreatic Cancer	Ineligible study design
**Wei et al. (2023)**	Factors influencing spiritual wellbeing among pancreatic ductal adenocarcinoma patients receiving chemotherapy	No data for prevalence
**Xia et al. (2022)**	Patient-caregiver dyads in pancreatic cancer: identification of patient and caregiver factors associated with caregiver well-being	No data for prevalence
**Yamaguchi et al. (2020)**	Health–related Quality of Life(QoL)in Japanese Patients with Cancer A Large–scale Questionnaire Survey Using EQ–5D–5L	No data for prevalence
**Yeo et al. (2021)**	Depression, rather than cancer-related fatigue or insomnia, decreased the quality of life of cancer patients	No data for PC
**Zhang et al. (2024)**	Application of nutrition management based on Patient-Generated Subjective Global Assessment in patients with pancreatic cancer undergoing chemotherapy	No data for prevalence
