# Supplementary material for: One out of four patients with pancreatic cancer experience psychological symptoms: A systematic review and meta-analysis
Source: PLoS One. 2026 May 27;21(5):e0348435. doi: 10.1371/journal.pone.0348435 (PMC13215498; doi:10.1371/journal.pone.0348435)
Supplement: S2 Table — Basic characteristics of the included studies in the meta-analysis. BDI-Beck Depression Inventory; BSI-Brief Symptom Inventory; CIDI-Composite International Diagnostic Interview; DT- Distress Thermometer EORTC QLQ-FA12 -European Organization for Research and Treatment of Cancer Fatigue; ESAS-Edmonton Symptom Assessment System; ESASr-Edmonton Symptom Assessment System Revised; EQ-5D VAS- EuroQol-5D- visual analogue scale; FACIT-G-Functional Assessment of Cancer Therapy – General; HADS-Hospital Anxiety and Depression Scale; HIS-Hornheider Screening Instrument; ICD-International Classification of Diseases; MMPI-Minnesota Multiphasic Personality Inventory; NCCN (DT)-National Comprehensive Cancer Network’s Distress Thermometer; PHQ-9-Personal Health Questionnaire; PO-Bado-Basic Documentation for Psycho-Oncology; PROMIS®-Patient-Reported Outcomes Measurement Information System; PSWQ-Penn State Worry Questionnaire; SAS-Self-rating Anxiety Scale; SCID-DSM-III-R-Structured Clinical Interview for Diagnostic and Statistical Manual of Mental Disorders; STAI-State Trait Anxiety Inventory. Time group: ‘short’-within six months from the diagnosis of cancer; ‘long’-beyond six months from the cancer diagnosis. NA-not applicable. (PDF) [file pone.0348435.s006.pdf]

First Author	Year of publication	Country	Mean (Age)	SD (Age)	Sex (%) female	Population size	Outcome	Measurement tool	Time group
Akizuki, et al.	2016	Japan	61.7	9.8	38.2	110	depression	DSM-III-R (SCID)	short
							anxiety		
Batra, et al.	2021	Canada	NA	NA	47.8	94	depression	ESASr	long
							anxiety		
Boyd, et al.	2012	USA	65.0	NA	45.5	22	depression	PHQ9	long
							anxiety	PSWQ	
Brintzenhofe, et al.	2009	USA	NA	NA	NA	185	depression	BDI	NA
							anxiety	HADS	
Carlson, et al.	2004	USA	NA	NA	NA	112	distress	BSI-18	short
Carlson, et al.	2019	USA	NA	NA	NA	148	distress	NCCN DT	short
Carruba, et al.	2022	Italy	NA	NA	NA	11	depression	HADS	NA
							anxiety		
Clark, et al.	2010	USA	60.4	10.8	45.1	304	depression	BSI-18	short
							anxiety		
							distress		
							fatigue	Problem Checklist	
Cui, et al.	2023	China	63.36	9.94	45.0	114	anxiety	EQ-5D VAS	NA
						134	fatigue		
Dai, et al.	2019	Canada	65.0	10.0	45.0	2043	distress	ESAS	short
Del Piccolo, et al.	2021	Italy	NA	NA	NA	400	depression	PHQ-9	short
							anxiety	STAI	
Fras, et al.	1967	USA	NA	NA	NA	50	depression	MMPI	short
							fatigue		
						35	anxiety		

Godby, et al.	2020	USA	70.0	7.2	43.7	88	depression	PROMIS®	short
Harris, et al.	2021	USA	NA	NA	NA	10378	depression<br>anxiety	NA	long
Hartung, et al.	2017	Germany	NA	NA	NA	82	depression	PHQ-9	short
Hohmann, et al.	2022	Germany	NA	NA	64	11	distress	HSI<br>PO-Bado	NA
Janda, et al.	2017	Australia	66.6	9.6	48.0	136	depression<br>anxiety	FACT G<br>HADS	long
Kim, et al.	2023	Canada	NA	NA	38.0	36	depression	PHQ-9	short
Lelond, et al.	2021	Canada	NA	NA	47.2	123	fatigue<br>anxiety	NA	short
Mehnert, et al.	2014	Germany	57.6	11.1	51.5	52	anxiety	CIDI	short
Pezzilli, et al.	2017	Italy	70.1	11.5	63.6	22	depression	BDI	short
Salm, et al.	2021	Germany	NA	NA	NA	80	depression<br>anxiety	From database	long
Schmidt, et al.	2020	Germany	NA	NA	NA	33	fatigue	EORTC QLQ-FA12	NA
Seoud, et al.	2020	USA	NA	NA	NA	10 220<br>62450<br>10 220	fatigue<br>depression<br>anxiety	ICD	long
Subramaniam, et al.	2024	USA	68	12.3	49.9	4029	depression<br>anxiety	From database	
Vehling, et al.	2022	Germany	58.6	10.98	49.5	50	depression<br>anxiety	CIDI	long
Yeo, et al.	2023	USA	69.4	34.9	46	403	depression<br>distress<br>fatigue	DT	NA
Zhang, et al.	2022	China	NA	NA	46	100	anxiety	SAS	NA
