# Supplementary material for: One out of four patients with pancreatic cancer experience psychological symptoms: A systematic review and meta-analysis
Source: PLoS One. 2026 May 27;21(5):e0348435. doi: 10.1371/journal.pone.0348435 (PMC13215498; doi:10.1371/journal.pone.0348435)
Supplement: S3 Table — Systematic review data table. BSI- Brief Symptom Inventory; PROMIS-Patient-Reported Outcomes Measurement Information System; NA-not applicable. (PDF) [file pone.0348435.s007.pdf]

First Author	Year of publication	Country	Mean (Age)	SD (Age)	Sex (%) female	Population size	Outcome	Measurement tool	Reason of exclusion from meta-analysis	Findings
Hussain, et al.	2023	Pakistan	NA	NA	NA	NA	depression anxiety	BSI	missing data	Depression: 55.97% Anxiety: 49.46 %
Jacobbson, et al.	1971	Sweden	66.0	NA	29.8	57	Depression anxiety sleep disorder	NA	information from relatives	Depression: 31.58% Anxiety: 12.28% Sleep disturbances: 21.05%
Mir, et al.	2020	England	70	7.1	44.3	64	cognitive complaints	PROMIS-SF-4a	not comparable data	Cognitive complaints: 29.69%
