# Supplementary material for: One out of four patients with pancreatic cancer experience psychological symptoms: A systematic review and meta-analysis
Source: PLoS One. 2026 May 27;21(5):e0348435. doi: 10.1371/journal.pone.0348435 (PMC13215498; doi:10.1371/journal.pone.0348435)
Supplement: S4 Table — N-no; Y-yes, U-unclear. The JBI appraisal checklist consists of 9 items and each item is assessed by scoring (yes = 1), (no = 0), and (unclear = 0). (PDF) [file pone.0348435.s008.pdf]

JBI Checklist No. ------------------------- First Author	1. Was the sample frame appropriate to address the target population?	2. Were study participants sampled in an appropriate way?	3.Was the sample size adequate?	4.Were the study subjects and the setting described in detail?	5.Was the data analysis conducted with sufficient coverage of the identified sample?	6.Were valid methods used for the identification of the condition?	7. Was the condition measured in a standard, reliable way for all participants?	8.Was there appropriate statistical analysis?	9.Was the response rate adequate, and if not, was the low response rate managed appropriately?	**Overall Score**	**Weight**
Akizuki, et al. 2016	Y	Y	N	Y	Y	Y	Y	Y	Y	8	88.8
Batra, et al. 2021	N	Y	N	N	Y	Y	Y	N	Y	5	55.5
Boyd et al. 2012	N	Y	N	Y	Y	Y	Y	Y	Y	7	77.7
Brintzenhofe, et al. 2009	Y	Y	Y	Y	U	Y	Y	Y	Y	8	88.8
Carlson, et al. 2004	Y	Y	Y	Y	Y	Y	Y	N	Y	8	88.8
Carruba, et al. 2022	Y	U	Y	Y	Y	Y	Y	Y	Y	8	88.8
Carlson, et al. 2019	Y	Y	Y	Y	Y	Y	Y	Y	Y	9	100
Clark, et al. 2010	Y	Y	N	Y	Y	Y	Y	Y	Y	8	88.8

JBI Checklist No. ------------------------- First Author	1. Was the sample frame appropriate to address the target population?	2. Were study participants sampled in an appropriate way?	3.Was the sample size adequate?	4.Were the study subjects and the setting described in detail?	5.Was the data analysis conducted with sufficient coverage of the identified sample?	6.Were valid methods used for the identification of the condition?	7. Was the condition measured in a standard, reliable way for all participants?	8.Was there appropriate statistical analysis?	9.Was the response rate adequate, and if not, was the low response rate managed appropriately?	**Overall Score**	**Weight**
Cui, et al. 2023	Y	Y	Y	Y	Y	Y	Y	Y	Y	9	100
Dai, et al. 2019	N	Y	Y	Y	Y	Y	Y	Y	Y	8	88.8
Del Piccolo, et al. 2021	N	Y	N	Y	N	Y	Y	Y	Y	6	66.6
Dengso, et al. 2020	Y	Y	Y	Y	Y	U	U	Y	Y	7	77.2
Fras, et al. 1967	Y	N	N	N	N	Y	Y	Y	Y	2	22.2
Godby, et al. 2020	Y	Y	Y	Y	N	Y	Y	Y	Y	8	88.8
Harris, et al. 2021	Y	Y	Y	Y	Y	U	U	Y	Y	7	77.7
Hartung, et al. 2017	Y	Y	N	Y	Y	Y	Y	Y	Y	8	88.8
Hohmann, et al. 2022	Y	Y	N	Y	Y	Y	Y	Y	Y	8	88.8

JBI Checklist No. ------------------------- First Author	1. Was the sample frame appropriate to address the target population?	2. Were study participants sampled in an appropriate way?	3.Was the sample size adequate?	4.Were the study subjects and the setting described in detail?	5.Was the data analysis conducted with sufficient coverage of the identified sample?	6.Were valid methods used for the identification of the condition?	7. Was the condition measured in a standard, reliable way for all participants?	8.Was there appropriate statistical analysis?	9.Was the response rate adequate, and if not, was the low response rate managed appropriately?	**Overall Score**	**Weight**
Janda, et al. 2017	Y	Y	N	Y	Y	Y	Y	Y	Y	8	88.8
Kim, et al. 2023	Y	Y	N	Y	Y	Y	Y	Y	Y	8	88.8
Lelond, et al. 2021	Y	Y	N	Y	N	Y	Y	U	Y	6	66.6
Mehnert, et al. 2014	Y	Y	Y	Y	Y	Y	Y	Y	Y	9	100
Pezzili, et al. 2017	N	Y	N	Y	N	Y	Y	Y	Y	6	66.6
Salm, et al. 2021	Y	Y	Y	N	Y	Y	Y	Y	Y	8	88.8
Schmidt, et al. 2020	Y	Y	Y	Y	Y	Y	Y	Y	Y	9	100
Seoud, et al. 2020	Y	Y	Y	Y	Y	Y	U	Y	Y	8	88.8
Subramaniam, et al. 2024	Y	Y	Y	Y	Y	Y	Y	Y	Y	9	100

**JBI Checklist No.** -------------------------- **First Author**	1. Was the sample frame appropriate to address the target population?	2. Were study participants sampled in an appropriate way?	3.Was the sample size adequate?	4.Were the study subjects and the setting described in detail?	5.Was the data analysis conducted with sufficient coverage of the identified sample?	6.Were valid methods used for the identificatio n of the condition?	7. Was the condition measured in a standard, reliable way for all participants ?	8.Was there appropriate statistical analysis?	9.Was the response rate adequate, and if not, was the low response rate managed appropriatel y?	**Overall Score**	**Weight**
Vehling, et al. 2022	Y	Y	Y	Y	Y	Y	Y	Y	Y	9	100
Yeo, et al. 2023	Y	Y	Y	Y	Y	Y	Y	Y	Y	9	100
Zhang, et al. 2022	Y	Y	Y	Y	Y	U	Y	Y	Y	7	88,8
