# Supplementary figures and images for: One out of four patients with pancreatic cancer experience psychological symptoms: A systematic review and meta-analysis
Source: PLoS One. 2026 May 27;21(5):e0348435. doi: 10.1371/journal.pone.0348435 (PMC13215498; doi:10.1371/journal.pone.0348435)

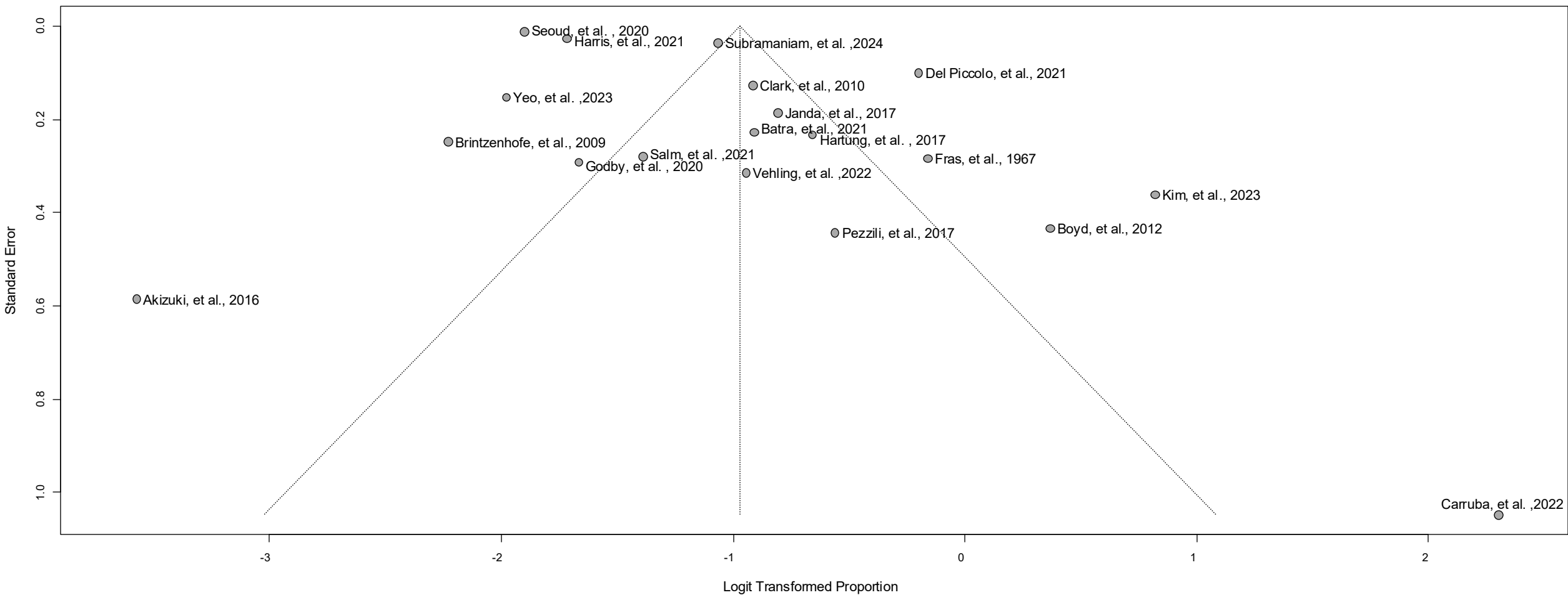

Supplement: S1 Fig — The points represent the different studies. It shows the residuals on the x-axis against their corresponding standard errors. Egger’s test p-value is 0.0413. (PDF) [file pone.0348435.s009.pdf]

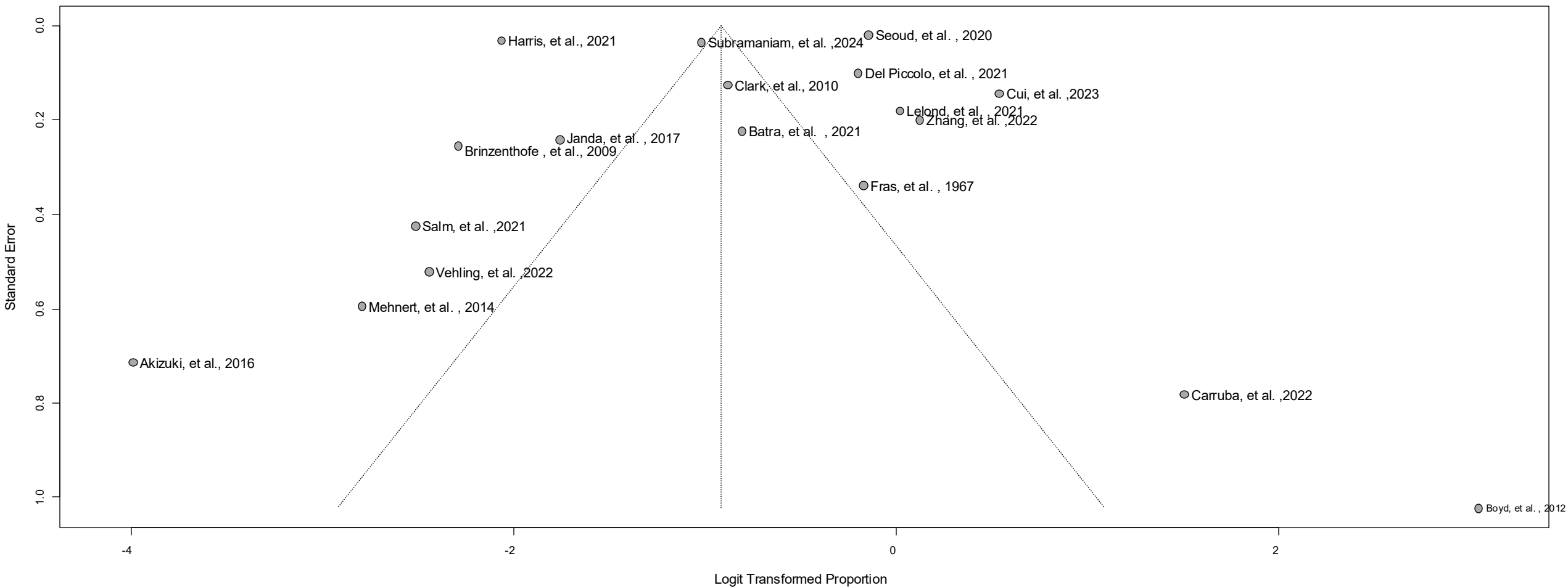

Supplement: S2 Fig — The points represent the different studies. It shows the residuals on the x-axis against their corresponding standard errors. Egger’s test p-value is 0.7877. (PDF) [file pone.0348435.s010.pdf]

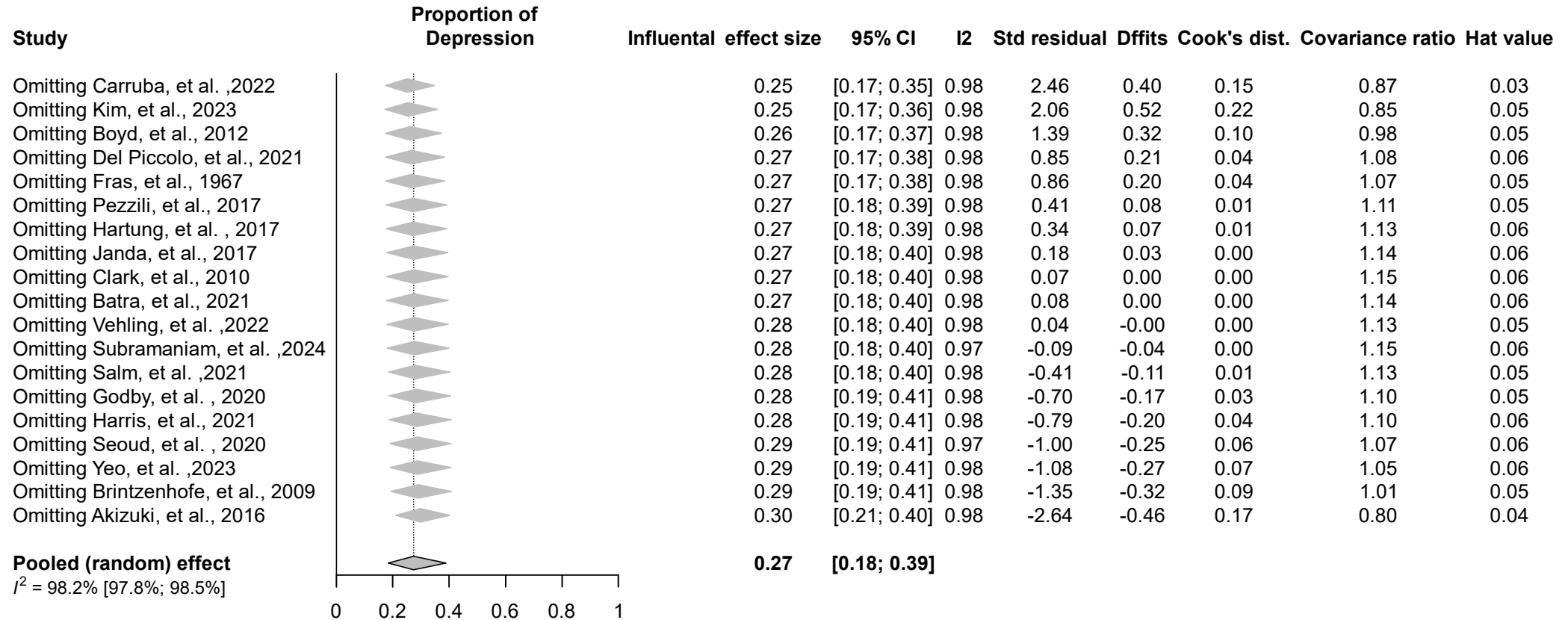

Supplement: S3 Fig — Figure shows how each individual study affects the overall estimate of the rest of the studies. (PDF) [file pone.0348435.s011.pdf]

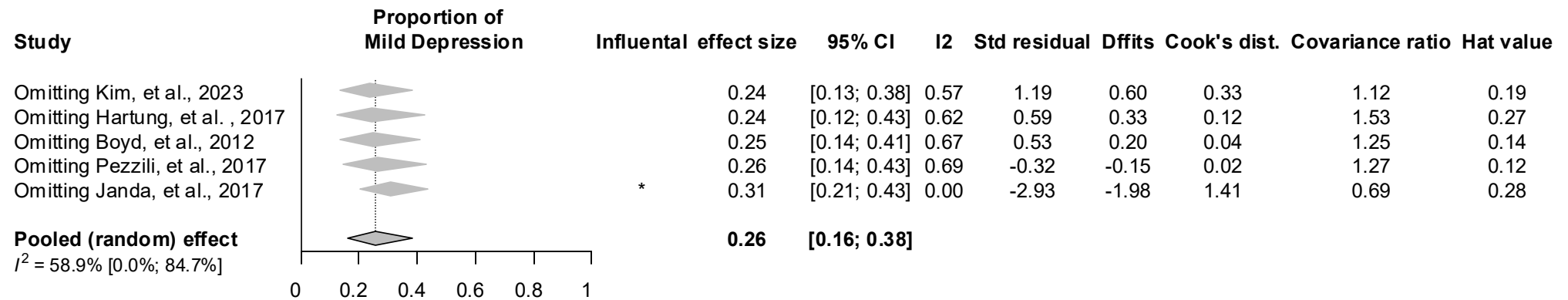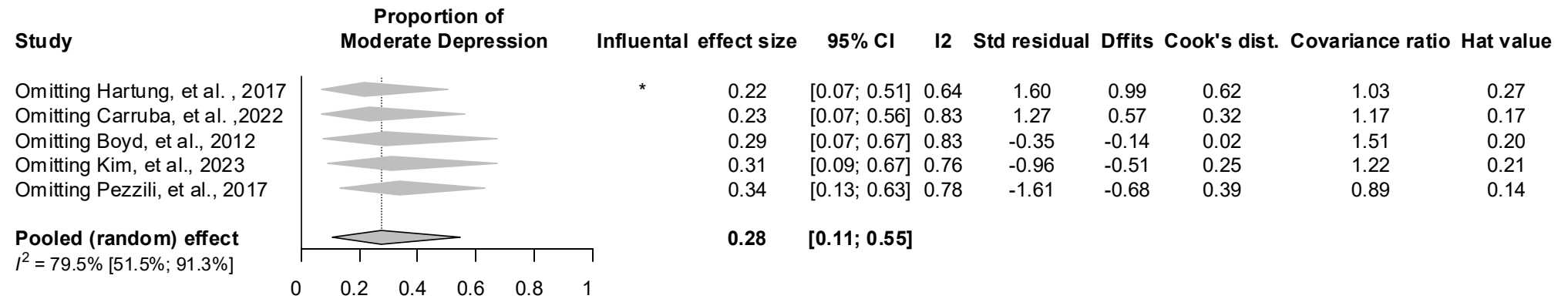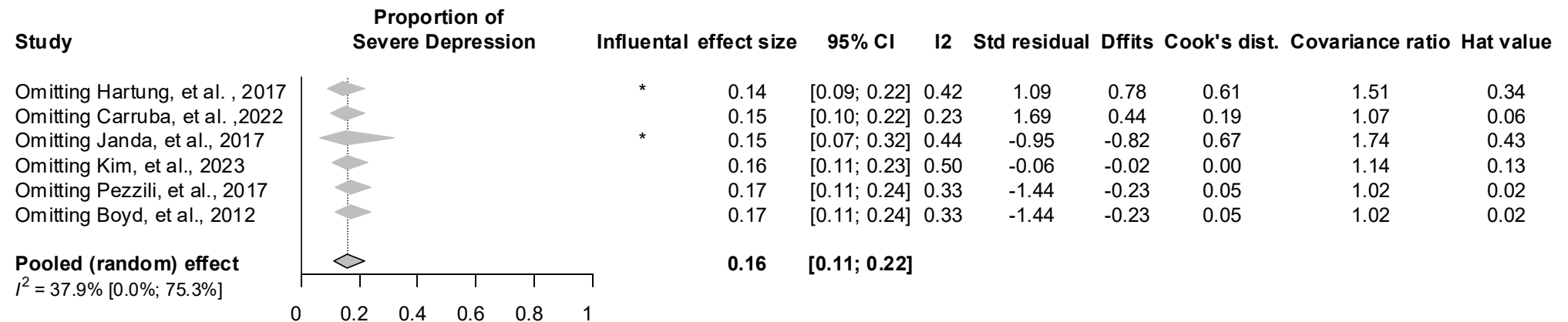

Supplement: S4 Fig — Figure shows how each individual study affects the overall estimate of the rest of the studies. (PDF) [file pone.0348435.s012.pdf]

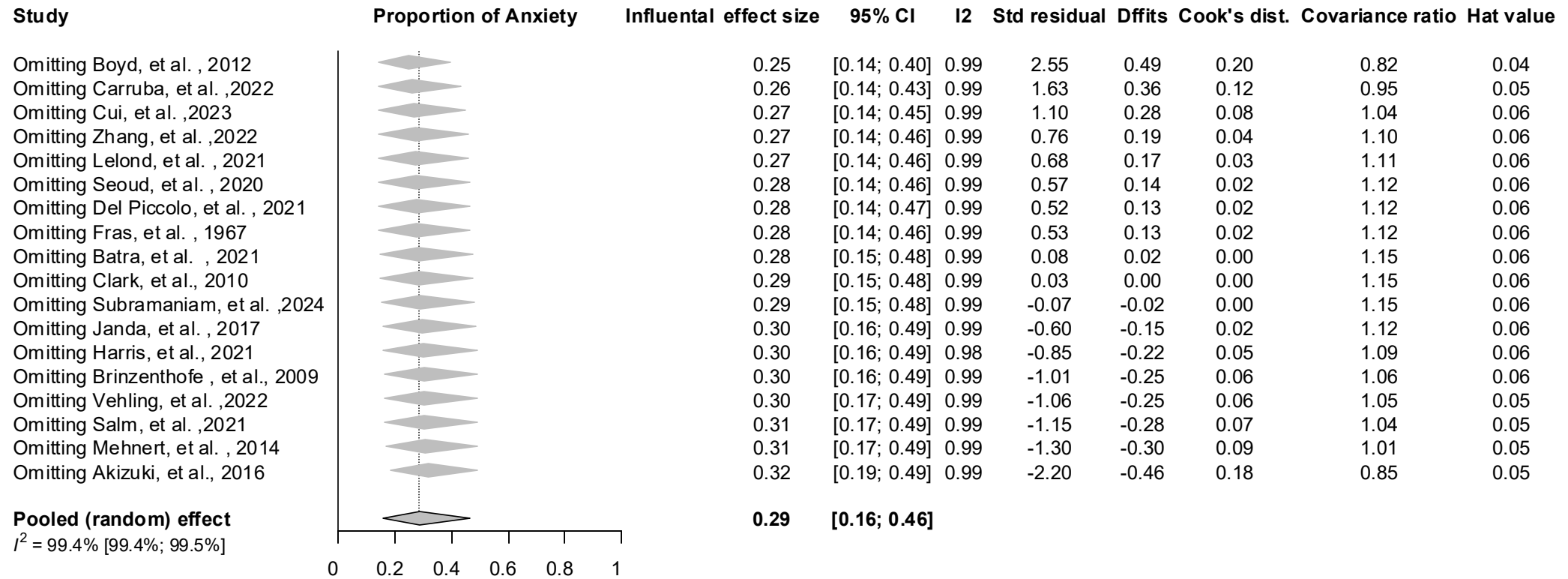

Supplement: S5 Fig — Figure shows how each individual study affects the overall estimate of the rest of the studies. (PDF) [file pone.0348435.s013.pdf]

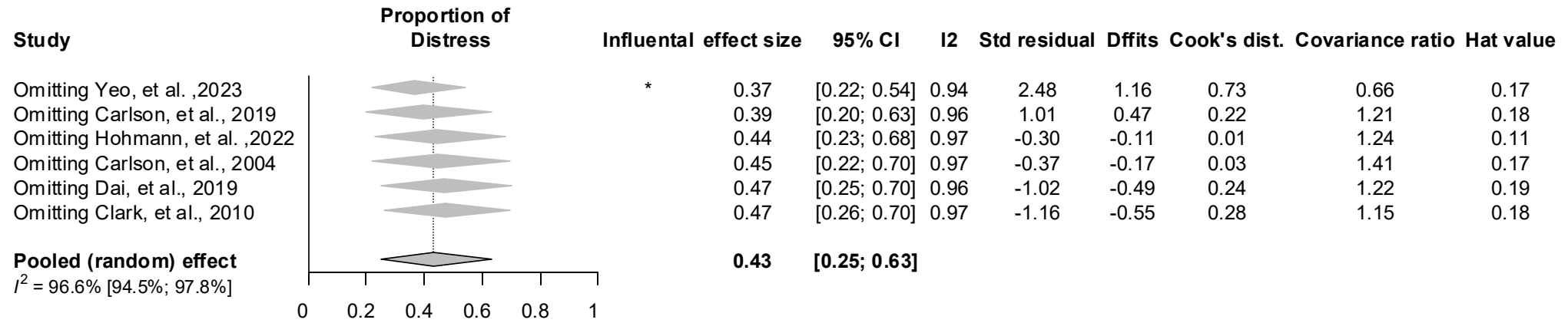

Supplement: S6 Fig — Figure shows how each individual study affects the overall estimate of the rest of the studies. (PDF) [file pone.0348435.s014.pdf]

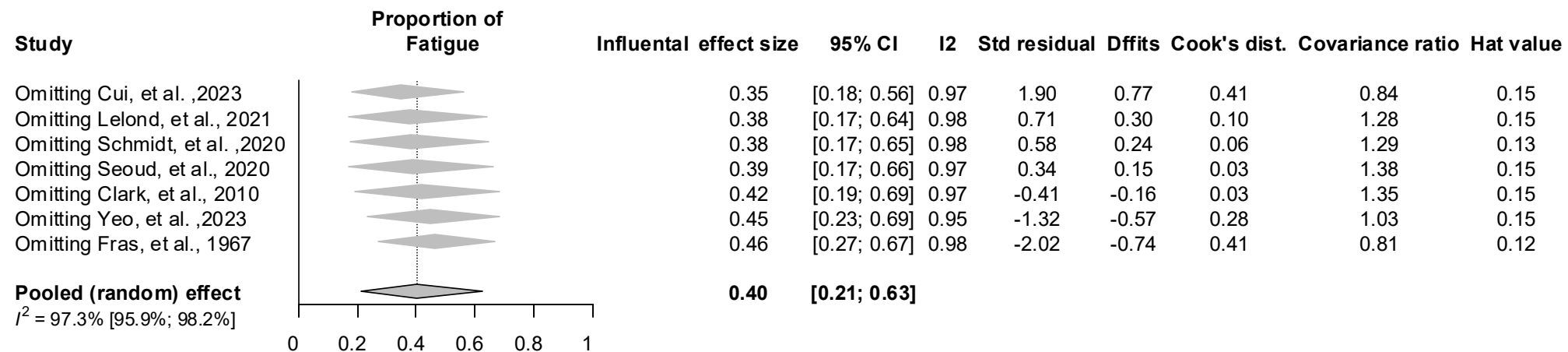

Supplement: S7 Fig — Figure shows how each individual study affects the overall estimate of the rest of the studies. (PDF) [file pone.0348435.s015.pdf]

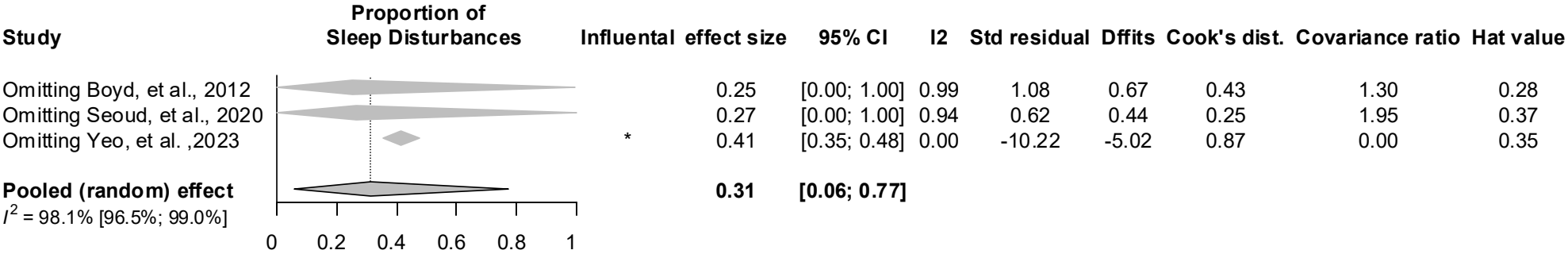

Supplement: S8 Fig — Figure shows how each individual study affects the overall estimate of the rest of the studies. (PDF) [file pone.0348435.s016.pdf]
