# Supplementary material for: One out of four patients with pancreatic cancer experience psychological symptoms: A systematic review and meta-analysis
Source: PLoS One. 2026 May 27;21(5):e0348435. doi: 10.1371/journal.pone.0348435 (PMC13215498; doi:10.1371/journal.pone.0348435)
Supplement: Attachment — Submitted filename: Response to Reviewers.pdf [file pone.0348435.s020.pdf]

Dear Editors,

Dr Cho Lee Wong,

Thank you for giving us the opportunity to submit a revised draft of our manuscript titled *One out of four patients with pancreatic cancer experience psychological symptoms: a systematic review and meta-analysis* to *PLOS ONE*. We appreciate the time and effort that you and the reviewers have dedicated to providing your valuable feedback on our manuscript. We are grateful to the reviewers for their insightful comments on our paper. We have been able to incorporate changes to reflect most of the suggestions provided by the reviewers.

We uploaded a marked-up copy of the manuscript that highlights changes made to the original version, labeled as 'Revised Manuscript with Track Changes'. As well as an unmarked version of the revised paper without tracked changes, labeled as ‘'Manuscript'.

In accordance with the editor's request, we have reviewed and updated the reference list. Several references were revised or replaced in the Introduction to reflect the most up-to-date statistical data, and additional references were added in the Discussion section to support the revisions made in response to the reviewers' comments. We confirm that no retracted articles are cited in the revised manuscript.

Here is a point-by-point response to the reviewers' comments and concerns.

Again, thank you for your very valuable time invested in our manuscript.

Best regards,

Péter Hegyi and his team.

Dear Reviewers,

Thank you very much for your valuable feedback. We greatly appreciated your comments which have significantly improved our manuscript. Please find below our point-by-point responses.

We hope that our revised manuscript will meet your expectations, and you will find it suitable for publication. Please let us know if any further changes are required.

Again, thank you for your very valuable time invested in our manuscript.

Best regards,

Péter Hegyi and his team.

Reviewer #3:

- **Comment 1:** "*1. Statistical Presentation of Proportions and Confidence Intervals*

  Although the authors have corrected the format of confidence intervals in the abstract (e.g., from "27%; CI=[0.18–0.39%]" to "0.27 (95% CI: 0.18–0.39)"), this correction has not been consistently applied throughout the manuscript. For instance, in the Results section (pp. 25–27), the same error persists (e.g., "proportion was 27%; [95% CI: CI=[0.18-0.39]%").

  Recommendation: Please ensure that all prevalence estimates and confidence intervals are reported consistently in the proportion format (e.g., 0.27, 95% CI: 0.18–0.39) rather than mixing percentages and proportions. This is essential for statistical accuracy and reader comprehension."

  Response 1: Thank you for this helpful observation. We agree that consistency in the statistical presentation of proportions and confidence intervals is essential for accuracy and readability.

  Action 1: Revised all instances where prevalence values were expressed as percentages (e.g., "27%") or mixed formats (e.g., "27%; 95% CI: 0.18–0.39%") to the standardized format: *0.27 (95% CI: 0.18–0.39)*.

- **Comment 2**: "*2. Clarity and Completeness of the Methods Section*

  2.1. The authors mention the use of the Joanna Briggs Institute (JBI) tool for quality assessment but do not provide a summary or interpretation of the results in the main text. The reader is referred to Supplementary Table S4, but a brief summary of the overall quality of included studies (e.g., how many were low, moderate, or high risk) should be included in the main manuscript to enhance transparency."

  Response 2.1: Thank you for the suggestion. We agree that including a concise summary of the JBI quality assessment results in the main text improves the clarity and transparency of the Results sections.

  Action 2.1: We have added the following sentence summarizing the overall quality of the included studies in the Results section, under *"Assessment of methodological quality"*. The paragraph now reads as follows:

  "*Assessment of methodological quality for evident synthesis*

  The quality of the included studies was assessed using the Joanna Briggs Institute Critical Appraisal Tool. Studies were grouped by risk of bias (high, medium, low), and these groupings are also reflected in the forest plots. Among the 28 included studies, 21 were rated as low risk of bias, six as moderate risk, and one as high risk according to the JBI tool. Detailed scores and assessment outcomes are provided in Supporting Information, Table S4."

- **Comment 2.2:** "*2. Clarity and Completeness of the Methods Section*

  The term "frequentist random-effects models" is used, but the rationale for choosing a frequentist over a Bayesian approach is not discussed. A brief justification would strengthen the methodology."

  Response 2.2: We appreciate the reviewer's thoughtful comment. While both Bayesian and frequentist frameworks are suitable for random-effects meta-analysis, we believe that the choice between them does not substantially affect the conclusions in this context. We therefore adopted the frequentist approach, which is the conventional and most commonly reported method in the literature. Since the vast majority of studies implicitly use this framework without explicitly stating it, we feel that a detailed discussion of the rationale in the Methods section would not substantially enhance the clarity or reproducibility of our analysis.

  Action 2.2: None.

- **Comment 3: "***3. Heterogeneity and Sensitivity Analyses*

  The I^2 values are exceptionally high (e.g., 98.2% for depression), indicating substantial heterogeneity. The authors briefly mention heterogeneity in the Discussion but do not sufficiently explore its potential sources (e.g., differences in measurement tools, cultural contexts, study populations).

  Recommendation: A more in-depth discussion of the possible reasons for heterogeneity is needed. Consider conducting subgroup or meta-regression analyses (if feasible) to explore sources of variation, such as geographic region, assessment tool, or time since diagnosis."

  Response 3: Thank you for this important observation. We also acknowledged that the I^2 values were exceptionally high across all examined outcomes, therefore we put a great effort on identifying key sources of heterogeneity as follows:

  Population Diversity, we wrote: "*The heterogeneity observed in the meta-analysis suggests that patient populations are diverse across studies. Differences in demographics, disease stages, treatment modalities, and geographic locations can impact reported values.*"

  Cultural Factors, we wrote: "*A more homogeneous population can provide a more accurate value for prevalence, as cultural and societal factors influence how individuals express and perceive psychological symptoms, as seen in the studies included with high or low prevalence, e.g., in the study by Akizuki et al., Variations in cultural norms, attitudes toward mental health, and the availability of support services can contribute to differences in reported proportions across studies.*"

  Assessment tool, we wrote: *"The measurement tools included in the studies are widely used in clinical and research settings ... Although they all offer standardized measures... they also raise questions of comparability and might affect the results." We further elaborated: "Variations in administration protocols, scoring methods, cultural*

adaptations, and severity across different studies can further complicate the comparability and interpretability of findings from these instruments."

We understand the need to further highlight the high heterogeneity observed across studies. We further made the existing points more direct and prominent to explain the sources of high heterogeneity.

We also agree that further exploration of heterogeneity would be beneficial. However, the available data were insufficient to perform subgroup or meta-regression analyses in a reliable and reliable way.

Action 3: We added: "*This analysis also confirms that the high observed heterogeneity might be multifactorial and not caused by a single influential outlier study*". The discussion of heterogeneity has been substantially expanded between **lines 309–362** (in the tracked-changes version of the manuscript). Additionally, we have revised the Limitations section to highlight the high heterogeneity observed across studies.

- **Comment 4: "** *Clinical and Policy Implications - The authors appropriately call for integrating psychological care into pancreatic cancer treatment. However, the recommendations remain general.*

  Recommendation: Provide more specific, actionable suggestions for clinicians and policymakers. For example: Routine screening for psychological symptoms using validated tools. Referral pathways to mental health services. Training for oncology care teams in basic psychological support."

  Response 4: Thank you for this constructive suggestion. We agree that providing more specific, actionable recommendations strengthens the clinical and policy implications of our findings.

  Action 4: We have revised the *Implications for practice, research and policymakers'* section to include more concrete strategies. Line 406-462 in the Manuscript with Tracked Changes file.

- **Comment 5:** *"Limitations Section. The limitations are mentioned briefly but should be expanded. Key issues include: High heterogeneity among studies. Potential underreporting of psychological symptoms due to stigma. Overrepresentation of certain geographic regions (e.g., Europe and North America), limiting generalizability. Variability in measurement tools and diagnostic thresholds"*

  Response 5: Thank you for your highlightings. We fully agree with your suggestion.

  Action 5: We added to the Limitation section the following:

  " [...]*These factors, together with cultural or language adaptations, differences in patient populations contribute to the high heterogeneity observed across analyses (I^2 consistently >90%), which should be considered when interpreting pooled prevalence estimates.*"

We believe further repetition is unnecessary, as these factors are already discussed in detail in the Discussion section.

- **Comment 6: "***Data Availability Statement. The current statement, "Data available in original articles," is not sufficient under PLOS ONE's data policy.*

  Recommendation: Please revise to state clearly that all data extracted for the meta-analysis are included in the manuscript and/or Supporting Information files. If data are only available from original studies, this must be explicitly stated with reasons."

  Response 6: We thank you for pointing this out. We agree that our original Data Availability Statement did not fully align with PLOS ONE's policy and have revised it accordingly for clarity and transparency.

  Action 6: We have updated the Data Availability Statement to the following: "*All data extracted for the meta-analysis are included in the manuscript tables, figures and Supporting Information files. A summarized excel table of all data is available for publicity upon request with reasons."*

- **Comment 7: "***Abstract: The sentence "Study quality was assessed using the Joanna Briggs Institute tool" is appropriate but should specify that this was done for included studies."*

  Response 7: Thank you for the recommendation.

  Action 7: We have revised the sentence to read: "*Study quality* ***for the included studies*** *was assessed using the Joanna Briggs Institute tool.*"

- **Comment 8: "***Figures: Ensure that all forest plots and figures are clearly labeled and that prediction intervals are included where appropriate."*

  Response 8: Upon careful review, all figures and captions appear to us to be clearly labeled. However, we would appreciate further clarification regarding which specific aspects were found unclear. With a bit more precision, we would be glad to make any additional adjustments or clarifications to ensure the figures fully meet the expectations.

  Regarding confidence intervals, Prediction intervals are already displayed in all figures where available, and this is explicitly noted in our manuscript: "*We summarized the findings from the meta-analysis in forest plots. We also reported the prediction intervals (i.e., the expected range of effects of future studies) of results, where applicable: the number of studies was large enough (>5), and the effects were not too heterogeneous."*

  Action 8: None.

- **Comment 9: "***References: Check consistency in reference formatting throughout the manuscript."*

Response 9: Thank you for pointing this out. We have carefully reviewed all references to ensure full consistency as authors - references with more than six authors contain *"et al."*, - title – Journal – year – additions

Action 9: All references have been rechecked and standardized.

Reviewer #4:

- **Comment 1: "***The authors should discuss more critically how differences in measurement tools may have influenced prevalence estimates."*

  Response 1: Thank you for this valuable comment. We have updated the Discussion section to provide a more critical reflection, including how differences in measurement tools and cut-off thresholds may have influenced the reported prevalence estimates.

  Action 1: The Discussion has been revised accordingly (lines 309–362 in the Revised Manuscript with Track Changes), and we hope that these additions meet your expectations.

- **Comment 2: "***Timing of symptoms onset, the study mentions " short vs. long" time from diagnosis but concludes timing does not affect outcomes. Please clarify."*

  Response 2: We thank you for this helpful comment and appreciate the opportunity to clarify this point. The "short" versus "long" categorization refers to the time elapsed since pancreatic cancer diagnosis, where "short" indicates within six months and "long" refers to beyond six months. These groupings were applied based on the data available in the included studies and are displayed in the forest plots for visual comparison. However, a formal statistical subgroup analysis was not conducted for this variable, as the number of studies providing time-specific data was limited and did not allow for a reliable quantitative comparison. Our conclusion that timing did not appear to affect outcomes was therefore based on visual inspection of the forest plots, which showed similar prevalence estimates between the "short" and "long" categories.

  Action 2: None.

- **Comment 3: "***The recommendation remain general not dependent on conclusion that called for psychological care with PC treatment."*

  Response 3: We have revised the *Implications for Practice, Research, and Policymakers* section to ensure that our recommendations are more specific and clearly aligned with the conclusion calling for the integration of psychological care into pancreatic cancer treatment.

  Action 3: These changes have been made starting from line 406 in the Revised Manuscript with Track Changes.

- **Comment 4:** **“***"Basic characteristics of studies included in the meta-analysis." please clarify why the 2 extra studies not included in mete-analysis.”*

  Response 4: Thank you for your comment. The reasons for excluding the three studies from the quantitative synthesis are presented in the Supplementary Materials (Table S3), which includes a dedicated “*Reason for exclusion from meta-analysis*” column. This table provides a comprehensive yet quick overview, allowing readers to access all relevant information efficiently. We believe this is the most transparent and practical way to present these details.

  Action 4: No changes have been made, as the information is already clearly and comprehensively presented in Supplementary Table S3. If you think that it is necessary. We would of course add an additional brief note in the main text.

- **Comment 5:** **“***Mention please how many studies for each categories(low, moderate, & sever) in all symptoms.”*

  Response 5: We thank you for this helpful suggestion. Data on severity were available only for depression, and we have now specified the number of studies contributing to each severity subgroup. For the other psychological symptoms (anxiety, distress, fatigue, and sleep disturbances), we didn’t have enough data, and therefore subgroup analysis by severity was not feasible.

  Action 5: We rewrote the section: *“In the studies where data based on severity was available the subset analysis based on severity showed that the proportion of mild depression was 0.26 (95% CI: 0.16-0.38), based on five studies; the proportion of moderate depression was 0.28 (95% CI: 0.11-0.55), based on five studies; and the proportion of severe depression was 0.16 (95% CI: 0.11-0.22), based on six studies. (Fig 2A.-2B.)”*

Reviewer #5:

- **Comment 1:** *“The manuscript is well-executed and addresses a critical issue in the management of pancreatic cancer patients. No revisions are needed, and the revisions made are thoughtful and improve the clarity and transparency of the study.*

  Therefore, I recommend accepting the manuscript without revision.”

  Response 1: We sincerely thank you for your positive feedback and kind words about our work. We are grateful for your time and consideration.

  Action 1: None

Reviewer #6:

- **Comment 1:** None
- **Response 1:** We thank you for your review and time dedicated to evaluating our manuscript.
- **Action 17:** None

Reviewer #7:

- **Comment 1:** *"Title: The title reads a bit like a research finding rather than a formal title. I suggest changing it to something like; "Psychological Symptoms in Pancreatic Cancer: Prevalence and Evidence from a Systematic Review and Meta-Analysis"*
- **Response 1:** We thank you for this thoughtful suggestion and fully understand the preference for a more neutral, formal title style. After careful consideration, and a meaningful discussion among authors, however, we decided to retain our original title, "Full Title: *One out of four patients with pancreatic cancer experience psychological symptoms: a systematic review and meta-analysis.* Short title*: Prevalence of psychological symptoms among patients with pancreatic cancer.*"

  We believe this title is specific, concise and comprehensible, which aligns with PLOS ONE's allowance for informative titles. Nevertheless, we are open to revising the title if the editor prefers a more formal version.
- **Action 1:** None.

- **Comment 2:** *In line 219, under the Distress subtitle, you mentioned "anxiety." I believe you intended to write "distress" instead. Kindly check and revise for accuracy.*
- **Response 2:** We sincerely thank you for pointing out this error. This was indeed a mistake on our part.
- **Action 2:** We have corrected line 219 under the Distress subtitle, replacing "anxiety" with "distress."

- **Comment 3:** **"***Early in the discussion, you repeat prevalence percentages already mentioned in the Results (lines 260–261, 267). Instead of re-listing numbers, focus on interpretation ("Our findings confirm that anxiety and depression are particularly common, with rates similar to or exceeding other cancers.").*
- **Response 3:** Very thoughtful suggestion, thank you. We understand the preference to focus on interpretation rather than repeating numerical results. However, we would like to retain the brief summary of prevalence values at the beginning of the Discussion, as it provides a concise orientation for readers who may not read the full Results section. In our experience, starting the Discussion by restating the main findings briefly offers a clear foundation for subsequent interpretation and contextualization.

- **Action 3:** No changes have been made, as we believe that the brief restatement of the key prevalence values strengthens the clarity and structure of the Discussion. We would be happy to adjust this section if the editor prefers a more interpretive opening.

- **Comment 4:** *"Be precise about definitions. Sometimes "distress" is used interchangeably with "anxiety," which may confuse readers. Define how distress was measured in your included studies."*

  Response 4: We thank you for this important comment. We would like to clarify that distress and anxiety were treated as distinct constructs and the terminology followed that of the original studies. As shown in 'Supplementary Table S2', each psychological outcome is listed with the specific measurement tool used. Distress was typically assessed with general emotional or psychosocial well-being instruments (e.g., Distress Thermometer, BSI-18, Edmonton Symptom Assessment System), while anxiety was measured with anxiety-specific scales (e.g., HADS-A, STAI, SAS). We acknowledge that in some instances within the Discussion, the term "distress" was used in a broader, non-technical sense to describe overall emotional strain; however, this does not reflect the analytic and technical distinction, which was maintained throughout the study.

  Action 4: None.

- **Comment 5:** **"***In line 347, instead of "as we know it today," use more formal phrasing: "To the best of our knowledge, this is the first ..."*

  Response 5: Thank you for pointing this out. We agree that "*To the best of our knowledge, this is the first …*" is a more formal and appropriate phrasing.

  Action 5: We have revised the sentence under "Strengths and Limitations" as follows:

  "*The strengths of our analysis, to the best of our knowledge, are that this is the first and most comprehensive meta-analysis on the topic, employs rigorous methods, and incorporates a high number of included patients and different psychological constructs in one article.*"

- **Comment 6:** **"***Under implications, be more specific about recommended practice: e.g., recommend routine screening for depression/anxiety/distress using validated tools (like HADS, Distress Thermometer)."*

  Response 6: We agree that providing more specific recommendations enhances the practical value of our implications.

  Action 6: Starting from line 406 in the Revised Manuscript with Track Changes, we have revised the section to provide more specific, actionable recommendations for practice, research, and policymakers, including routine screening using validated tools, structured interviews, evidence-based psychological interventions, and the need for trained psychologists.

- **Comment 7:** ”*For policy implications, suggest strategies: e.g., inclusion of psycho-oncology in national cancer care guidelines, training oncology staff in psychological screening.”*

  Response 7: Thank you for this suggestion. We agree that including specific policy-level strategies strengthens the relevance and applicability of our recommendations.

  Action 7: We have updated the policy implications to recommend that policymakers support research, ensure dedicated psycho-oncology teams in oncology wards, and implement policies to guarantee equitable access to psycho-oncological services. Starting from line 406 in the Revised Manuscript with Track Changes.

- **Comment 8:** ”*For research implications, highlight the need for interventional trials more to test effectiveness of psychological support in PC patients.”*

  Response 8: Thank you for your suggestion.

  Action 8: We have revised the *Implications for practice, research and policymakers section* starting from line 406 in the Revised Manuscript with Track Changes.

- **Comment 9:** ”*In line 382, consider tightening wording: "psychological alterations" → "psychological symptoms" (for more consistency with earlier sections).”*

  Response 9: We appreciate this insightful suggestion. We agree that "psychological symptoms" provides greater consistency with the terminology used in earlier sections.

  Action 9: We have revised line 382, replacing "psychological alterations" with "psychological symptoms."

Reviewer #8:

- **Comment 1:** ”*Update of Statistics – In the Introduction, the prevalence and mortality numbers for pancreatic cancer are based on 2023 reports. Since this is a 2025 submission, the authors should cite the most recent epidemiological data using Siegel 2025 Cancer Statistics to ensure accuracy.”*

  Response 1: Thank you so much for this insightful recommendation, we fully agree with your suggestion. We have now incorporated the most recent statistics to ensure accuracy.

  Action 1: We have revised the "Introduction":

- **Comment 2:** ”*Link to Survival Outcomes – The Introduction highlights the psychological burden but does not explicitly connect these symptoms to clinical outcomes. The authors should mention that depression, anxiety, and distress can negatively affect treatment*

adherence, quality of life, and survival rates in pancreatic cancer patients. Including this would strengthen the rationale."

Response 2: We thank you for this helpful suggestion. The impact of psychological symptoms on clinical outcomes is already discussed in our Discussion section:

"*This knowledge gap is particularly critical given the potential negative implications of psychological factors on disease course[18], poorer survival, and higher cancer-specific mortality.[69]*").

We agree that briefly mentioning this in the Introduction strengthens the rationale for the study.

Action 2: We added the following to the Introduction:

"*Research has demonstrated that elevated levels of depression, anxiety and distress can negatively impact quality of life, predict poorer adherence to treatment regimens, and are independently associated with decreased overall survival rates in this patient population.*"

- **Comment 3:** "*Figures/Tables – Figure 1 (PRISMA diagram) should be redrawn in higher resolution. Some table footnotes should be expanded to explain abbreviations and statistical notations more clearly."*

  Response 3: Thank you. We understand the importance of high-quality figures and clear table footnotes for readability. To ensure we address the concern accurately, we would appreciate clarification on which abbreviations or statistical notations require further explanation.

  Action 3: Figure 1 (PRISMA diagram) has been checked using the Preflight Analysis and Conversion Engine (PACE) tool, which confirmed that the resolution meets PLOS ONE requirements.

- **Comment 4:** "*Measurement Tools – Multiple instruments were used across studies (e.g., HADS, PHQ-9, BDI). A comparative table showing cut-off scores and interpretability would help clarify variability across the tools."*

  Response 4: Although cut-off scores for validated instruments (e.g., HADS, PHQ-9, BDI) varied across studies, we have discussed their influence on prevalence and heterogeneity in the text, even more detailed now based on other suggestions. Including a detailed table of cut-offs for each study would provide limited additional insight, because heterogeneity arises from multiple sources beyond cut-off thresholds, such as differences in study populations, disease stage, treatment modalities, cultural contexts, and questionnaire administration. Of course, if you think that such a table adds significant value to interpretation, we would include it; however, we believe that the discussion already effectively conveys the main factors driving variability in prevalence estimates.

  Action 4: None.

- **Comment 5:** **"***Publication Bias – Funnel plots were only presented for depression and anxiety. The authors should clearly acknowledge the limitation that other outcomes could not be assessed due to smaller sample sizes."*

  Response 5: Thank you for your suggestion. We agree that it is necessary to explicitly acknowledge this limitation.

  Action 5: We have updated the "Strengths and Limitations" section with the following:

  "Additionally, funnel plots were only presented for depression and anxiety; other outcomes could not be assessed due to smaller sample sizes, which may limit the evaluation of potential publication bias for these outcomes."

- **Comment 6:** **"***Clinical Implications – The conclusion currently recommends "targeted interventions," but this is too broad. The authors should propose more concrete strategies such as: Routine psychological screening at diagnosis, or Integration of psycho-oncology referrals."*

  Response 6: Thank you for this valuable suggestion. We agree that the recommendation for "targeted interventions" was too broad and required greater specificity.

  Action 6: We have revised the *Implications for practice, research and policymakers section* to include more concrete strategies, starting from line 406 in the Revised Manuscript with Track Changes.

We would like to thank you once again for all the reviewers' time and thoughtful comments. Your feedback has been invaluable in helping us to strengthen the manuscript. We look forward to hearing from you in due time regarding our submission and to respond to any further questions and comments you may have.

Sincerely,

Péter Hegyi and his team.
