# Supplementary material for: One out of four patients with pancreatic cancer experience psychological symptoms: A systematic review and meta-analysis
Source: PLoS One. 2026 May 27;21(5):e0348435. doi: 10.1371/journal.pone.0348435 (PMC13215498; doi:10.1371/journal.pone.0348435)
Supplement: Attachment — Submitted filename: Response_to_Reviewer.pdf [file pone.0348435.s021.pdf]

Dear Dr Cho Lee Wong,

Thank you for the opportunity to submit a revised version of our manuscript. *One out of four patients with pancreatic cancer experience psychological symptoms: a systematic review and meta-analysis* to *PLOS ONE*. We sincerely appreciate the time and effort you and the reviewers have dedicated to providing constructive feedback. We have carefully considered all of your comments and incorporated revisions to address the suggestions provided.

We uploaded a marked-up copy of the manuscript that highlights changes made to the original version, labeled as 'Revised Manuscript with Track Changes'.

As well as, an unmarked version of the revised paper without tracked changes, labeled as ''Manuscript'.

Here is a point-by-point response to the reviewer's comments and concerns.

Again, thank you for your very valuable time invested in our manuscript.

Best regards,

Péter Hegyi and his team.

- **Comment 1:** "*Abstract. Interpretation of pooled estimates: Given the very high heterogeneity (I^2 >90%), the Abstract should be more cautious in presenting point estimates without qualification.*"

  Response 1: We thank you for this suggestion and agree that the high level of heterogeneity requires cautious interpretation. However, due to the PLOS ONE abstract word limit and multiple revisions already implemented in response to previous reviewer comments, we were unable to further modify the Abstract without exceeding the permitted length. We believe that the implications of the very high heterogeneity are explicitly discussed later in the manuscript.

  Action 1: No changes were made to the Abstract. If you still think that a qualification in the Abstract is necessary, we would be happy to revise it accordingly. Please advise on which part of the abstract to remove in order to not exceed the word limit.

- **Comment 2**: "*Introduction. The Introduction is well written and convincingly justifies the need for this systematic review. A brief early clarification distinguishing psychological symptoms from diagnosed psychiatric disorders would further strengthen the conceptual framing.*"

  Response 2: Thank you for your comment. We have added an explicit clarification in the Introduction part as well stating that this review focuses on psychological symptoms assessed by screening measures rather than on clinically diagnosed psychiatric disorders, and we have integrated this distinction into the stated aim of the meta-analysis.

  Action 2: We have added the following: "*This meta-analysis aims to consolidate available evidence and quantify the burden of psychological symptoms in patients*

with PC, as assessed by screening measures rather than clinically diagnosed psychiatric disorders."

- **Comment 3:** *"Method: Search strategy: The search was conducted in MEDLINE, EMBASE, and Cochrane Library on May 22, 2022,119 and updated on March 3, 2025. There was no date or language restriction. How did you deal with the different language papers?"*

  Response 3: Thank you for pointing this out.

  Action 3: We added the following sentence to address this point: […]for non-English articles, when none of the co-authors were fluent in the language, we used online translation tools to assess eligibility and extract relevant data.

- **Comment 4:** *"Eligibility Criteria: While the heterogeneity of psychological constructs was appropriately acknowledged, the Methods section does not clearly distinguish between clinically diagnosed psychiatric disorders and screening-based symptom measures. Explicit clarification that the outcomes represent psychological symptom burden rather than diagnostic prevalence would substantially improve methodological transparency."*

  Response 4: We thank you for this comment and agree that an explicit clarification improves methodological transparency.

  Action 4: We added the following sentence: "*For the purposes of this review, outcomes were conceptualized as psychological symptom burden rather than prevalence of clinically diagnosed psychiatric disorders."*

- **Comment 5:** *"Statistical Analysis: Given the substantial methodological and conceptual heterogeneity across included studies, the authors should more explicitly justify the decision to pool prevalence estimates derived from different outcome definitions."*

  Response 5: We thank the reviewer for this important comment. We fully acknowledge the substantial methodological and conceptual heterogeneity across the included studies (e.g.measurement tools). Despite these differences, we decided to pool prevalence estimates because all included outcomes were intended to capture the same underlying clinical construct. Although assessed using different instruments, the instruments used across studies were conceptually aligned and aimed to identify individuals meeting a comparable clinical or functional threshold rather than fundamentally distinct conditions.We explored the possibility of subgroup analyses; however, the available data were insufficiently detailed and too heterogeneous to form subgroups (except depression severity).

  Action 5: We added the following sentence to the Discussion : "*The pooled prevalence should be interpreted as an average estimate across heterogeneous operational definitions rather than a precise prevalence for any single measurement approach.*"

- **Comment 6:** *"Result: Study Characteristics: Fig 1. PRISMA Flowchart. Please provide reasons for excluding studies. A brief clarification in the main text explaining why certain studies were excluded from quantitative synthesis would aid readers."*

  Response 6: Thank you for your suggestion, we agree with it.

  Action 6: We made the following changes to the main text: "*After assessing the full text, we excluded 194 articles, mainly due to ineligible study design (n = 12), no data on pancreatic cancer outcomes (n = 32), or no prevalence data (n = 44), resulting in 28 papers being used in the meta-analysis and 31 in the systematic review section.*"

- **Comment 7:** *"Heterogeneity and Publication Bias: The implications of I^2 values exceeding 90% could be more clearly emphasised in the Results, not only in the Discussion."*

  Response 7: Thank you for your comment. We agree that it needs to be more emphasised in the Result section too.

  Action 7: We added the following sentence to our Result section: "*High heterogeneity was observed across studies, with I^2 values exceeding 90% for all measured outcomes, indicating substantial variability between studies.*"

We thank you again for your constructive comments. We look forward to your feedback and are happy to address any further questions or clarifications

Best regards,

Péter Hegyi and his team.
