# Supplementary material for: One out of four patients with pancreatic cancer experience psychological symptoms: A systematic review and meta-analysis
Source: PLoS One. 2026 May 27;21(5):e0348435. doi: 10.1371/journal.pone.0348435 (PMC13215498; doi:10.1371/journal.pone.0348435)
Supplement: Attachment — Submitted filename: Bognar_et_al._Manuscript track change.docx [file pone.0348435.s017.docx]

**Full Title**

**One out of four patients with pancreatic cancer experience psychological symptoms: a systematic review and meta-analysis**

**Short title**

**Prevalence of psychological symptoms among patients with pancreatic cancer.**

**Authors**

Anna Sára Bognár^1^, Dóra Andrea Sükös^1^, Cristina Patoni^1,2^, Eszter Ágnes Szalai^1,3^, Brigitta Teutsch^1,4,5^ Dániel Sándor Veres^1,6^, Nóra Hosszúfalusi^1,7^, Bálint Mihály Erőss^1,5,8^ Katalin Márta^1,8,9&^ and Péter Hegyi^1,5,8,9,&,*^

^&These authors contributed equally to this work.^

**Affiliations:**

1. Centre for Translational Medicine, Semmelweis University, Budapest, Hungary
2. Carol Davila University of Medicine and Pharmacy, Bucharest, Romania
3. Department of Restorative Dentistry and Endodontics, Semmelweis University, Budapest, Hungary
4. Department of Radiology, Medical Imaging Centre, Semmelweis University, Budapest, Hungary
5. Institute for Translational Medicine, Szentágothai Research Centre, Medical School, University of Pécs, Pécs, Hungary
6. Department of Biophysics and Radiation Biology, Semmelweis University, Budapest, Hungary
7. Department of Internal Medicine and Hematology, Semmelweis University, Budapest, Hungary
8. Institute of Pancreatic Diseases, Semmelweis University, Budapest, Hungary
9. Translational Pancreatology Research Group, Interdisciplinary Centre of Excellence for Research Development and Innovation, University of Szeged, Szeged, Hungary

***Corresponding author:**

E-mail: [hegyi2009@gmail.com](mailto:hegyi2009@gmail.com) (PH)

**ABSTRACT**

Pancreatic cancer has one of the highest mortality rates among cancers, with rising incidence globally. Despite its poor prognosis, the psychological burden including depression, anxiety, and distress remains understudied. We aimed to quantify the prevalence of these symptoms to inform patient centred care.

This systematic review and meta-analysis followed the preferred reporting items for systematic Reviews and Meta-Analyses guidelines. The study protocol was registered with PROSPERO under record number CRD42022288805. We conducted a systematic search (PROSPERO: CRD42022288805) of MEDLINE, Cochrane, and Embase databases to identify articles reporting on the prevalence of psychological symptoms among patients with pancreatic cancer. The symptoms examined were depression, anxiety, distress, fatigue, and sleep disturbances. A meta-analysis of the proportion of symptoms with a 95% confidence interval (CI) was conducted using frequents random-effects models. Subset analyses were performed based on the severity of depression. Example (The risk of bias was assessed using a funnel plot and Egger’s test. The results were presented using tables, figures, and statements).

.A total of 28 articles were included in the meta-analysis and 31 in the systematic review. The pooled prevalence estimates were depression 27% (95% CI: 18-39%; 19 studies, n=78,930), anxiety 29% (16-46%; 18 studies, n=26,538), distress 43% (25-63%; 6 studies, n=2,746), fatigue 40% (21-63%; 7 studies, n=11,338), and sleep disturbances 31% (6-77%; 3 studies, n=10,645).

most and least effective tr The prevalence of psychological symptoms among pancreatic cancer patients is strikingly high, with contributing factors including depression, anxiety, distress fatigue and sleep disturbances. Accordingly, healthcare systems should implement integrated psychological services that provide routine psychological assessment and address the unique emotional needs of these patients.

Keywords: pancreatic neoplasm, mental health, oncology

# INTRODUCTION

Pancreatic cancer (PC) is currently the third deadliest cancer type in the United States, with 64,050 new cases diagnosed in 2023, leading to the loss of 50,550 lives [1]. The prevalence of PC has been steadily increasing, making it a substantial public health concern[2], and by 2040, the estimated number of incidents is expected to almost double.[3] The same trend can be seen across Europe [4]. This will pose significant clinical challenges and have profound psychological implications for patients, their families, and healthcare professionals.

It has been shown earlier that psychological interventions have beneficial effects on patients with cancer and can improve their quality of life at the onset of their disease [5]. Despite these findings, the International Psycho-Oncology Society (IPOS)[6] has found significant differences in the delivery of psycho-oncology services. In Europe, 40–60% of patients with cancer and their families face psychological distress that could be treated with adequate interventions. However, only a minority of individuals in this group receive the psychological support and care they need.[7] Indeed, only 37% of European countries have an allocate budget for psycho-oncological support, and in most Eastern European countries, mental health remains a low priority. On the contrary, addressing psychological issues are still stigmatized, and their impact is overlooked.[8]

As the prevalence of PC increases, mental health problems need to be addressed even more, and we need to map patients' psychological needs to personalize and provide the best possible care. Although individual studies may have previously reported on the psychological symptoms of patients with PC, there is still a need for a comprehensive overview that can initiate future research, clinical practice, and interventions in this specific oncological context. This meta-analysis aims to consolidate available information and quantify the degree of psychological symptoms among patients with PC.

# METHODS

For this systematic review and meta-analysis, we followed the recommendations of the Cochrane Collaboration and Preferred Reporting Items for Systematic Reviews and Meta-Analyses (PRISMA) reporting guidelines.[9] The PRISMA checklist can be found in the Supporting Information, Table 1 (Table S1). The review protocol was registered at PROSPERO (CRD42022288805) and no deviation was necessary.

This systematic review and meta-analysis was conducted in line with guidelines outlined in the Preferred Reporting Items for Systematic Reviews and Meta-Analysis (PRISMA) (9). The PRISMA checklist can be found in the Supporting Information, Table 1 (Table S1). The study protocol was registered in the International Prospective Register of Systematic Reviews (PROSPERO) under registration number CRD42022288805 and no devation was necessary.

## Eligibility criteria

Our clinical question was what is the prevalence of psychological symptoms among patients with pancreatic cancer? We formulated our clinical question and defined eligibility criteria for our review using the CoCoPop (Condition, Context, Population) framework. We included full articles reporting the prevalence of any psychological symptoms among adult patients with diagnosed pancreatic ductal adenocarcinoma, regardless of stage. Given that the term 'psychological symptoms' is a vague term widely used in the literature, any definition was accepted. The definitions of depression, anxiety, distress, fatigue, and sleep disturbances varied across studies and were often vague. We accepted them if they were based on standardized diagnostic criteria, or if they are commonly used in clinical and research settings with their specified cut-off scores in the articles. For the subgroup analysis on severity, we used the categorization of the original articles (mild, moderate, severe). The exclusion criterion was any premorbid psychiatric disease. Case reports and case series were not considered eligible; however, any other type of article was eligible.

## Search strategy

The search was conducted in MEDLINE, EMBASE, and Cochrane Library on May 22, 2022, and updated on March 3, 2025. There was no date or language restriction. The search key can be found in in the Supporting Information in Documentum S1. We reviewed the reference lists of identified relevant studies to see if any additional relevant studies might be eligible for our studies.

## Selection process

Duplicates were removed using reference manager software (EndNote X9, Clarivate Analytics, Philadelphia, PA, USA). Titles, abstracts, and, later, full texts were selected by two independent authors in pairs (DAS and CP) based on pre-specified criteria. Any possible disagreements were resolved by a third party (SAB). Cohen's kappa coefficients were calculated to measure inter-rater reliability.

## Data collection process

Two authors (DAS and SAB) independently collected data from the eligible articles manually into a preformed, standardized data table (Microsoft Excel, Microsoft Office 365, Redmond, WA, USA). Disagreements were resolved by a third person (CP).

## Data items

The following data were extracted: publication characteristics: first author, year of publication, country of origin, number of centers, demographic characteristics of the study population, measurement tool, time of measurement (referred to as time groups), occurrence of psychological symptoms as reported in each article. Prevalence was estimated as the proportion of PC patients affected by a psychological symptom at any given time.

## Study risk of bias assessment

Two independent reviewers (DAS and SAB) assessed the methodological quality of the studies using The Joanna Briggs Institute Prevalence Critical Appraisal Tool.[10] A third reviewer (CP) resolved potential disagreements about the risk of bias scoring. The tool includes nine questions on study design, duration of the study, retention of participants, and study results. A 'yes' option is provided to indicate appropriate methodological quality. Alternatively, a 'no' option can be selected if poor quality is indicated or 'unclear' for ambiguous responses, improving the assessment of study quality. Each item is assessed using a score (yes = 1), (no = 0), and (unclear = 0). The total score from each study was also presented as a percentage. Different levels of risk of bias are categorized as follows: high risk of bias = 20–50% items scored yes; moderate risk of bias = 50–80% items scored yes; and low risk of bias = 80–100% items scored yes, based on the JBI checklist.

## Effect measures and synthesis methods

As we assumed considerable between-study heterogeneity in all cases, a random-effects model was used to pool effect sizes in a frequentist method. The proportion (prevalence) of symptoms with a 95% confidence interval (CI) was used for effect size measure. The total number of patients and those with the event of interest were extracted from each study to calculate the proportion. We summarized the findings from the meta-analysis in forest plots. We also reported the prediction intervals (i.e., the expected range of effects of future studies) of results, where applicable: the number of studies was large enough (>5), and the effects were not too heterogeneous. Between-study heterogeneity was described by the between-study variance (τ^2^) and Higgins and Thompson’s I^2^ statistics.[11] Results were reported in the format of effect size or statistics and [95% CI]. Small study publication bias was assessed by visual inspection of funnel plots and calculating modified Egger’s test p-values. However, we kept in mind that the test had limited diagnostic assessment below ~10 studies. Potential outlier publications were detected using different influence measures and plots following the recommendation by Harrer et al.[12] All statistical analyses were calculated by R software[13] (v4.3.3) using the meta[14] (v7.0.0) package for basic meta-analysis calculations and plots and the dmetar[15] (v0.1.0) package for additional influential analysis calculations and plots. For more details on data synthesis methods and influential analysis, see ‘Data synthesis – detailed methods' in the Supporting Information, in Documentum S2.

# RESULTS

## Search and selection

Altogether, 23,282 studies were identified using our search key. After duplicate removal and exclusions based on titles and abstracts, 313 papers were screened for eligibility. After assessing the full text, we excluded 194 articles, which resulted in 28 papers being used in the meta-analysis and 31 in the systematic review section. The whole process is described in the PRISMA flowchart Fig 1.

**Fig 1. PRISMA Flowchart.**

## Basic characteristics of studies included in the meta-analysis.

The baseline characteristics of the analyses included are detailed in Table S2. In summary, 19 studies[16-31] reported on depression, 18 studies[16-19, 21-23, 26, 27, 30-37] on anxiety, 6 studies[21, 29, 38-41] on distress, 7 studies[18, 21, 27, 29, 32, 37, 42] on fatigue, and three studies on sleep disturbances [18, 29, 31]. The number of participants ranged from 11 to 62,450. The baseline characteristics of the three additional studies[43-45] in the systematic review are detailed in Table S3. Their reported results showed similar prevalence to the ones in the meta-analysis.

| Frist auhor | Study priod | Design | Country | Setting | Type (optional) | Event | Total participant |
| --- | --- | --- | --- | --- | --- | --- | --- |
| Schwarzer, G.,2023 |  |  |  |  |  | More of events positive or interest of researchers. example yes or no depression so yes 3000 no 2000 | Total yes/no depression 5000 |

## The quality score of the study

## The quality of the included study was evaluated using the ____________ Studies having an_____score of ____and above were included in the final analysis (Reference). (Table).

Example if TABLE(-) Newcastle‐Ottawa quality assessment scales of the eligible studies.

Example if use Newcastle

| **S/N** | **First author** | **NOS criteria** | | | **Total (10)** | **Remark** |
| --- | --- | --- | --- | --- | --- | --- |
|  |  | Selection (5) | Comparability (2) | Outcome (3) |  |  |
| 1 | Schwarzer, G.,2023(reference) | **** | - | *** | 7 | Included |

## Prevalence of psychological symptoms among patients with PC

### Depression

For depression, 78,930 patients from 19 studies were involved in our analysis. Our results showed that the proportion was 27%; CI = [0.18-0.39%]; I^2^ = 98,2% [97,8%-98,5%]. In the studies where data based on severity was available the subset analysis based on severity showed that the proportion of mild depression was 26%; CI = [0.16-0.38%]; the proportion of moderate depression was 28%; CI = [0.11-0.55%]; and the proportion of severe depression was 16%; CI = [0.11-0.22%]. (Fig 2A.-2B.)

**Fig 2A.-2B.** **Prevalence of depression.** The forest plot shows the prevalence of depression total (2A) following a pancreatic cancer diagnosis using subgroup analysis based on severity (2B). ‘Short’-within six months of cancer diagnosis; ‘long’-beyond six months from cancer diagnosis. ROB- Risk of bias, CI-Confidence interval

### Anxiety

Eighteen studies involving 26,538 patients were included in our analysis of anxiety. Our results showed that the proportion of patients with anxiety was 29%; CI = [0.16-0.46%]; I^2^ = 99,4% [99,4%-99,5%]. (Fig 3.)

**Fig 3.** **Prevalence of anxiety.** The forest plot shows the prevalence of anxiety following a diagnosis of pancreatic cancer. ‘Short’-within six months of cancer diagnosis; ‘long’-beyond six months from cancer diagnosis. ROB- Risk of bias, CI-Confidence interval

### Distress

Data from 2746 patients from six studies were analyzed for anxiety. Our results showed that the proportion of distress was 43%; CI = [0.25-0.63%]; I^2^ = 96,6% [94,5%-97,8%]. (Fig 4.)

**Fig 4.** **Prevalence of distress.** The forest plot shows the prevalence of distress following a diagnosis of pancreatic cancer. ‘Short’-within six months of cancer diagnosis. ROB- Risk of bias, CI-Confidence interval

### Fatigue

Seven studies reporting fatigue with 11,338 patients were involved in our analysis. Our results showed that the prevalence was 40%; CI = [0.21-0.63%]; I^2^ = 97,3% [95,9%-98,2%]. (Fig 5.)

**Fig 5.** **Prevalence of fatigue.** The forest plot shows the prevalence of fatigue following a diagnosis of pancreatic cancer. ‘Short’-within six months of cancer diagnosis; ‘long’-beyond six months from cancer diagnosis. ROB- Risk of bias, CI-Confidence interval

### Sleep disturbances

Three studies reporting sleep disturbances with 10,645 patients were involved in our analysis. Our results showed that the prevalence was 31%; CI = [0.06-0.77%]; I^2^ = 98,1% [96,5%-99,0%]. (Fig 6.)

**Fig 6.** **Prevalence of sleep disturbances.** The forest plot shows the prevalence of sleep disturbances following a diagnosis of pancreatic cancer. ‘Short’-within six months of cancer diagnosis; ‘long’-beyond six months from cancer diagnosis. ROB- Risk of bias, CI-Confidence interval

## Assessment of methodological quality

The outcomes of the evaluation process conducted by the two reviewers are self-explanatory and visualized in a table. (Table S4).

## Add Sensitivity analysis

Clearly

## Publication bias and heterogeneity

We provided the funnel plot analysis with p-values for depression and anxiety only (Figures S1-2), as we were not given a funnel plot assessment when the study number was below ten. Egger’s test p-value is 0.0413 for depression indicating potential publication bias. Egger’s test p-value is 0.7877 for anxiety indicating no potential publication bias. The Leave-one-out analysis revealed that the potential influential studies have no relevant impact on the pooled result (Figures S3-8).

# DISCUSSION

The purpose of this systematic review and meta-analysis was to investigate the prevalence of psychological symptoms among patients with PC. Our findings showed that the examined outcomes developed in more than a quarter of the studied population (depression 27%, anxiety 29%, distress 43%, fatigue 40%, and sleep disturbances 31%). Although we were not able to compare the effect of time from diagnosis (categorized as 'short'—within six months of cancer diagnosis, and 'long'—beyond six months from cancer diagnosis), the forest plots suggest that the timing of diagnosis does not impact the development of these psychological symptoms in a relevant amount, allowing plenty of time for interventions.

Mood disturbances, especially depression and anxiety, are often presenting complaints in patients with PC, with an overall prevalence ranging from 30% to 50%.[46] This study indicates a significant occurrence of **depression** among patients with PC. Earlier studies have found that 30% of patients with PC report symptoms of clinical depression.[21, 47] Our subset analysis of depression based on severity reveals that prevalence is variable. A potential reason for the lower prevalence in the severe group is the rapid deterioration of the patient's condition. These patients often require heavy medication, including sedation, to reduce symptom burden, particularly when admitted to the intensive care unit. Similarly, our results indicate that a substantial proportion of patients experience **anxiety** alongside their cancer diagnosis. Even in early-stage disease, anxiety is common before surgery and preoperative counseling.[46] In a systematic review comparing anxiety and depressive symptoms in adults with other cancers, patients with PC showed greater anxiety and depressive symptoms.[48]

Sleep disturbance is a common symptom in patients with cancer. [49] Sleep disturbance and fatigue data specific to the population with PC are rare, but most studies report high fatigue levels at presentation and throughout treatment.[50] Our findings show that fatigue affects a significant portion of the population. Treatments for PC often lead to loss of skeletal muscle mass, which can increase fatigue and psychological distress.[51] The proportion of **distress** was also high in our findings and is similar to earlier reports in which psychological distress affected approximately 40% of patients diagnosed with PC.[47, 52]

Previous studies have suggested that symptoms of fatigue, anxiety, and depression are strongly interconnected.[53, 54] Compared with healthy adults or population norms, adults with pancreatic cancer experienced a lower quality of life (QoL) in all domains. Additionally, when compared with patients with other types of cancer, those with PC reported poorer psychological QoL, with a significant strain on psychological well-being.[48]

Understanding the complexity of underlying factors is crucial when interpreting prevalence. The real-life presence of psychological symptoms among patients reported in the meta-analysis may be even higher for many reasons. Psychological symptoms, such as depression, anxiety, and distress, might be underreported due to stigma or reluctance to address mental health issues.[55] Cancer patients, including those with pancreatic cancer, may be hesitant to acknowledge or seek help for emotional challenges, which may lead to an underestimation of the prevalence of the symptoms.

The heterogeneity observed in the meta-analysis suggests that patient populations are diverse across studies. Differences in demographics, disease stages, treatment modalities, and geographic locations can impact reported values. A more homogeneous population can provide a more accurate value for prevalence, as cultural and societal factors influence how individuals express and perceive psychological symptoms, as seen in the studies included with high or low prevalence, e.g., in the study by Akizuki et al.[16] Variations in cultural norms, attitudes toward mental health, and the availability of support services can contribute to differences in reported proportions across studies. Age is also an important factor for patients with PC. The risk for distress in patients with cancer was almost double that of the general population.[56] However, studies found that older age groups (61 years and above) showed almost no differences in psychological distress between patients with cancer and the general population, with differences being higher in younger patients.[56, 57] Even though PC is typically the disease of the elderly, the incidence is growing among the younger population.[58-60]

The measurement tools included in the studies are widely used in clinical and research settings. Information on the questionnaire per study can be found in Table S2. Although they all offer standardized measures for assessing various psychological constructs such as depression, anxiety, distress, and fatigue, they also raise questions of comparability and might affect the results.[61] Variations in administration protocols, scoring methods, cultural adaptations, and severity across different studies can further complicate the comparability and interpretability of findings from these instruments.[62, 63] The goal should be to standardize the definition of these constructs and narrow the use of these measurement tools to achieve more subjective and comparative results in psychological research.

Previous studies[64-66] have highlighted the critical role of psychological factors on cancer recurrence and mortality in different types of cancer. However, our understanding of the psychological burden associated with pancreatic cancer remains limited compared to other types of cancer, perhaps because less attention has been paid to it. A recent study[67] has revealed that many common and deadly cancers, including pancreatic cancer, receive the least research funding relative to their prevalence and mortality. Conversely, breast cancer and pediatric cancers are well-funded despite their lower mortality. These findings align with previous research, including an analysis of European cancer research investment from 2016 to 2020, highlighting the disproportionate research.[68]

A first step toward development in this area is to assess the presence and extent of psychological burdens in PCs. This knowledge gap is particularly critical given the potential negative implications of psychological factors on disease course[18], poorer survival, and higher cancer-specific mortality.[69]

The relatively high occurrence of psychological symptoms in this study underscores the importance of integrating psychological care into the comprehensive treatment of pancreatic cancer. Treating patients with cancer should be a multidisciplinary approach, where the aim is not only to treat physical symptoms but psychological ones as well, as they can influence the course and outcome of the disease. Disparities in access in many countries and smaller towns remain challenging[8] - leaving patients, their families, and healthcare professionals struggling with the emotional complexities of these serious cases without support. Bridging these gaps in access to psychological care is not only imperative for the well-being of individual patients, but also a fundamental step toward improving the holistic quality of care for those facing the challenges of pancreatic cancer.

By providing a detailed synthesis of existing data, our findings not only fill a critical gap in the literature and could serve as a base for further research but also advocate for increased funding and resources dedicated to understanding and addressing the burdens faced by patients with PC.

## Strengths and limitations

The strengths of our analysis, as we know it today, are that this is the first and most comprehensive meta-analysis on the topic, employs rigorous methods, and incorporates a high number of included patients and different psychological constructs in one article.

As for the limitations, besides the low number of studies on each psychological symptom, differences in the assessment tools and criteria used across studies can impact the prevalence. The articles' definitions of symptoms were variable and vague, measured with different tools, and severity thresholds varied.

## Implications for practice, research and policymakers

Addressing psychological symptoms, including depression, anxiety, distress, fatigue, and sleep disturbances is necessary for holistic patient care, and the translation of new scientific findings into clinical practice is crucial.[70, 71] The findings of this study, which reveal a relatively high prevalence of psychological symptoms (depression, anxiety, distress, and fatigue) among patients with pancreatic cancer, underscore the critical need to integrate psychological support and interventions into the comprehensive care of these patients. This is particularly significant when considered alongside our previous research, which has demonstrated that psychological interventions can substantially enhance the QoL for patients, especially with early-stage cancer.[5] By combining the insights from our earlier work with the current findings, we strongly advocate for the routine incorporation of psychological care into cancer treatment protocols, not only for early-stage patients but also for those facing the severe challenges of pancreatic cancer. Evidence suggests that addressing psychological symptoms is not just an additional aspect of care but an essential component that can influence treatment outcomes, patient well-being, and overall quality of life. There is an urgent need for standardized methods to assess psychological symptoms across cancer types, as well as policies to ensure equitable access to psycho-oncological services. This integrated approach will better meet the diverse needs of cancer patients, improving their survival and holistic health outcomes.

Our results also highlight further research opportunities for targeted interventions, such as psychoeducation, counseling, and psychotherapy, which can be developed based on a deeper understanding of these factors. The burden of age-related symptoms may be an exciting area for further investigation. Longitudinal studies can explore how psychological symptoms evolve and change over time, considering treatment phases and post-treatment experiences, as the disease stage can greatly impact the outcomes.

# CONCLUSION

The high prevalence of psychological alterations, including depression, anxiety, distress, fatigue, and sleep disturbances emphasizes the need for comprehensive psychological care as a core component of pancreatic cancer treatment.

## Acknowledgments

None to declare.

## Data availability:

Data available in the original articles.

## Conflict of interest:

None to declare.

## Ethical approval

No ethical approval was required for this systematic review with meta-analysis, as all data were already published in peer-reviewed journals. No patients were involved in the design, conduct or interpretation of our study.

Datasets used in this study can be found in the full-text articles included in the systematic review and meta-analysis.

## Supporting Information

Supporting Information with the following content submitted in one document.

**Documentum S1. Search Key**

**Documentum S2. Data synthesis – detailed methods**

**Table S1. PRISMA checklist.** From: Page MJ, McKenzie JE, Bossuyt PM, Boutron I, Hoffmann TC, Mulrow CD, et al. The PRISMA 2020 statement: an updated guideline for reporting systematic reviews. BMJ 2021;372: n71. doi: 10.1136/bmj. n71

**Table S2. Basic characteristics of the included studies in the meta-analysis. Basic characteristics of the included studies in the meta-analysis.** BDI-Beck Depression Inventory; BSI-Brief Symptom Inventory; CIDI-Composite International Diagnostic Interview; DT- Distress Thermometer EORTC QLQ-FA12 -European Organization for Research and Treatment of Cancer Fatigue; ESAS-Edmonton Symptom Assessment System; ESASr-Edmonton Symptom Assessment System Revised; EQ-5D VAS- EuroQol-5D- visual analogue scale; FACIT-G-Functional Assessment of Cancer Therapy - General; HADS-Hospital Anxiety and Depression Scale; HIS-Hornheider Screening Instrument; ICD-International Classification of Diseases; MMPI-Minnesota Multiphasic Personality Inventory; NCCN (DT)-National Comprehensive Cancer Network’s Distress Thermometer; PHQ-9-Personal Health Questionnaire; PO-Bado-Basic Documentation for Psycho-Oncology; PROMIS®-Patient-Reported Outcomes Measurement Information System; PSWQ-Penn State Worry Questionnaire; SAS-Self-rating Anxiety Scale; SCID-DSM-III-R-Structured Clinical Interview for Diagnostic and Statistical Manual of Mental Disorders; STAI-State Trait Anxiety Inventory. Time group: ‘short’-within six months from the diagnosis of cancer; ‘long’-beyond six months from the cancer diagnosis. NA-not applicable

**Table S3. Basic characteristic table of additional included articles that were not meta-analyzed. Systematic review data table.** **BSI**- Brief Symptom Inventory; **PROMIS**-Patient-Reported Outcomes Measurement Information System; **NA**-not applicable

**Table S4. The Joanna Briggs Assessment of the methodological quality.** N-no; Y-yes, U-unclear. The JBI appraisal checklist consists of 9 items and each item is assessed by scoring (yes = 1), (no = 0), and (unclear = 0). Weight: high risk of bias = 20–50% items scored yes; moderate risk of bias = 50–80% items scored yes; and low risk of bias = 80–100% items scored yes.

**Figure S1. Funnel plot analysis of depression.** The points represent the different studies. It shows the residuals on the x-axis against their corresponding standard errors. Egger’s test p-value is 0.0413.

**Figure S2. Funnel plot analysis of anxiety.** The points represent the different studies. It shows the residuals on the x-axis against their corresponding standard errors. Egger’s test p-value is 0.7877.

**Figure S3. Leave-one-out analysis of depression.** Figure shows how each individual study affects the overall estimate of the rest of the studies.

**Figure S4. Leave-one-out analysis of the subset of depression.** Figure shows how each individual study affects the overall estimate of the rest of the studies.

**Figure S5. Leave-one-out analysis of anxiety.** Figure shows how each individual study affects the overall estimate of the rest of the studies.

**Figure S6. Leave-one-out analysis of distress.** Figure shows how each individual study affects the overall estimate of the rest of the studies.

**Figure S7. Leave-one-out analysis of fatigue.** Figure shows how each individual study affects the overall estimate of the rest of the studies.

**Figure S8. Leave-one-out analysis of sleep disturbances.** Figure shows how each individual study affects the overall estimate of the rest of the studies.

Add

Author’s contribution:

Abbreviation and acronyms (optional) but I see in abstract like PC, PRISMA………………………………………………………

# REFERENCES

1.	*National Cancer Institute*

*Cancer of the pancreas - cancer stat facts. The Surveillance, Epidemiology, and End Results (SEER)*. 2024; Available from: <https://seer.cancer.gov/statfacts/html/pancreas.html>.

2.	Siegel, R.L., et al., *Cancer statistics, 2023.* CA Cancer J Clin, 2023. **73**(1): p. 17-48.

3.	*Cancer Tomorrow. Global Cancer Observatory*. 2024 2024.02.08. [cited 2024 2024.04.27.]; Available from: https://gco.iarc.fr/tomorrow/en/dataviz/isotype?cancers=13&types=0&mode=population&group_populations=0&multi p le_populations=0&multiple_cancers=0&single_unit=50000.

4.	Partyka, O., et al., *Overview of Pancreatic Cancer Epidemiology in Europe and Recommendations for Screening in High-Risk Populations.* Cancers (Basel), 2023. **15**(14).

5.	Bognár, S.A., et al., *Psychological intervention improves quality of life in patients with early-stage cancer: a systematic review and meta-analysis of randomized clinical trials.* Sci Rep, 2024. **14**(1): p. 13233.

6.	Travado, L., et al., *2015 President's Plenary International Psycho-oncology Society: psychosocial care as a human rights issue-challenges and opportunities.* Psychooncology, 2017. **26**(4): p. 563-569.

7.	European Cancer Organization. *Patient Insights on Cancer Care: Opportunities for Improving Efficiency*. 2019 July 2019 [cited 2024 April]; Available from: <https://www.europeancancer.org/resources/61:news-patient-insights-on-cancer-care-opportunities-for-improving-efficiency.html>.

8.	Coppini, V., et al., *Disparities and barriers in the assessment of psychological distress, access to and use of psycho-oncological support in Europe: current perspectives.* Front Psychol, 2023. **14**: p. 1252843.

9.	Higgins JPT, T.J., Chandler J, Cumpston M, Li T, Page MJ, Welch VA, *Cochrane Handbook for Systematic Reviews of Interventions. 2nd Edition*. 2019: Chichester (UK): John Wiley & Sons, 2019.

10.	Munn Z, M.S., Lisy K, Riitano D, Tufanaru C., *Methodological guidance for systematic reviews of observational epidemiological studies reporting prevalence and incidence data.* Vol. Int J Evid Based Healthc. 2015;13(3):147–153. 2015: The Joanna Briggs Institute.

11.	Higgins, J.P. and S.G. Thompson, *Quantifying heterogeneity in a meta-analysis.* Stat Med, 2002. **21**(11): p. 1539-58.

12.	Harrer, M., et al., *Doing Meta-Analysis with R: A Hands-On Guide*. 2021.

13.	R Core team, *A Language and Environment for Statistical Computing. Vienna, Austria: R Foundation for Statistical Computing.* 2022.

14.	Schwarzer, G., *Schwarzer, G. Meta: General Package for Meta-Analysis*. 2023.

15.	Ebert, M.H.a.P.C.a.T.F.a.D.D., *Cuijpers P, Furukawa T, Ebert DD. Dmetar: Companion R Package For The Guide 'Doing Meta-Analysis in R*. 2019.

16.	Akizuki, N., et al., *Prevalence and predictive factors of depression and anxiety in patients with pancreatic cancer: A longitudinal study.* Japanese Journal of Clinical Oncology, 2016. **46**(1): p. 71-77.

17.	Brintzenhofe, K.M., et al., *Mixed anxiety/depression symptoms in a large cancer cohort: Prevalence by cancer type.* Psychosomatics, 2009. **50**(4): p. 383-391.

18.	Seoud, T., et al., *Depression before and after a Diagnosis of Pancreatic Cancer: Results from a National, Population-Based Study.* Pancreas, 2020. **49**(8): p. 1117-1122.

19.	Harris, J.P., et al., *Longitudinal analysis of mental disorder burden among elderly patients with gastrointestinal malignancies.* JNCCN Journal of the National Comprehensive Cancer Network, 2021. **19**(2): p. 163-171.

20.	Godby, R., et al., *Depression among older adults with gastrointestinal (GI) malignancies: Results from the Cancer and Aging Resilience Evaluation (CARE) registry.* Journal of Clinical Oncology, 2020. **38**(4).

21.	Clark, K.L., et al., *Psychological distress in patients with pancreatic cancer - An understudied group.* Psycho-Oncology, 2010. **19**(12): p. 1313-1320.

22.	Batra, A., et al., *Symptom burden in patients with common cancers near end-of-life and its associations with clinical characteristics: a real-world study.* Supportive Care in Cancer, 2021. **29**(6): p. 3299-3309.

23.	Janda, M., et al., *Anxiety, depression and quality of life in people with pancreatic cancer and their carers.* Pancreatology, 2017. **17**(2): p. 321-327.

24.	Hartung, T.J., et al., *The risk of being depressed is significantly higher in cancer patients than in the general population: Prevalence and severity of depressive symptoms across major cancer types.* European Journal of Cancer, 2017. **72**: p. 46-53.

25.	Pezzilli, R., A. Cucchetti, and L. Calculli, *Comparison of Clinical Data and Scores of Quality of Life, Anxiety, and Depression in Patients with Different Types of Intraductal Papillary Mucinous Neoplasms: A Prospective Study.* Pancreas, 2017. **46**(8): p. 1029-1034.

26.	Del Piccolo, L., et al., *Prevalence of depression in a cohort of 400 patients with pancreatic neoplasm attending day hospital for major surgery: role on depression of psychosocial functioning and clinical factors.* Psycho-oncology, 2021. **30**(4): p. 455‐462.

27.	Fras, I. and E.M. Litin, *Comparison of psychiatric manifestations in carcinoma of the pancreas, retroperitoneal malignant lymphoma, and lymphoma in other locations.* Psychosomatics, 1967. **8**(5): p. 275-7.

28.	Kim, C.A., et al., *The impact of early palliative care on the quality of life of patients with advanced pancreatic cancer: The IMPERATIVE case-crossover study.* Support Care Cancer, 2023. **31**(4): p. 250.

29.	Yeo, T.P., et al., *Distress, Depression, and the Effect of ZIP Code in Pancreaticobiliary Cancer Patients and Their Significant Others.* J Am Coll Surg, 2023. **236**(2): p. 339-349.

30.	Carruba, G., et al., *Unmet Nutritional and Psychological Needs of Cancer Patients: An Integrated Multi-Professional Model Approach.* Diseases, 2022. **10**(3).

31.	Boyd, A.D., et al., *Screening for depression, sleep-related disturbances, and anxiety in patients with adenocarcinoma of the pancreas: A preliminary study.* The Scientific World Journal, 2012. **2012**.

32.	Lelond, S., et al., *Symptom burden of patients with advanced pancreas cancer (APC): A provincial cancer institute observational study.* Current Oncology, 2021. **28**(4): p. 2789-2800.

33.	Salm, S., et al., *Mental disorders and utilization of mental health services in newly diagnosed cancer patients: An analysis of German health insurance claims data.* Psycho-Oncology, 2021. **30**(3): p. 312-320.

34.	Vehling, S., et al., *Prevalence of mental disorders in patients with cancer compared to matched controls–secondary analysis of two nationally representative surveys.* Acta Oncologica, 2022. **61**(1): p. 7-13.

35.	Subramaniam, D.S., et al., *Understanding the relationship between palliative care consultation and mental health services in patients with pancreatic cancer: A US cross-sectional analysis.* Journal of Clinical Oncology, 2024. **42**(16).

36.	Zhang, L., et al., *Influence of continuing nursing care team mode on the rehabilitation of patients with pancreatic cancer after postoperative daily chemotherapy.* Support Care Cancer, 2022. **30**(11): p. 9003-9009.

37.	Cui, L., et al., *Translation and validation of the Pancreatic Cancer Disease Impact score for Chinese patients with pancreatic cancer: A methodological and cross-sectional study.* Asia Pac J Oncol Nurs, 2023. **10**(4): p. 100209.

38.	Dai, W.F., et al., *Are population-based patient-reported outcomes associated with overall survival in patients with advanced pancreatic cancer?* Cancer Medicine, 2020. **9**(1): p. 215-224.

39.	Carlson, L.E., et al., *High levels of untreated distress and fatigue in cancer patients.* Br J Cancer, 2004. **90**(12): p. 2297-304.

40.	Carlson, L.E., et al., *Prevalence of psychosocial distress in cancer patients across 55 North American cancer centers.* Journal of Psychosocial Oncology, 2019. **37**(1): p. 5-21.

41.	Hohmann, L., et al., *Assessment and Evaluation of Psychosocial Distress in Outpatients with Cancer at a University Hospital in Germany.* Oncology Research and Treatment, 2023. **46**(10): p. 424-432.

42.	Schmidt, M.E., et al., *Prevalence and severity of long-term physical, emotional, and cognitive fatigue across 15 different cancer entities.* Cancer Medicine, 2020. **9**(21): p. 8053-8061.

43.	Hussain, M., et al., *A RESEARCH STUDY ON THE INCIDENCE OF PSYCHOLOGICAL STRESS IN A SIGNIFICANT NUMBER OF PEOPLE WITH CANCER.* NeuroQuantology, 2023. **21**(2): p. 37-41.

44.	Jacobsson, L. and J.O. Ottosson, *Initial mental disorders in carcinoma of pancreas and stomach.* Acta Psychiatr Scand Suppl, 1971. **221**: p. 120-7.

45.	Mir, N., et al., *Patient-reported cognitive complaints in older adults with gastrointestinal malignancies at diagnosis- Results from the Cancer & Aging Resilience Evaluation (CARE) study.* J Geriatr Oncol, 2020. **11**(6): p. 982-988.

46.	Safyan, R.A., et al., *Multidisciplinary Standards and Evolving Therapies for Patients With Pancreatic Cancer.* American Society of Clinical Oncology Educational Book, 2024. **44**(3): p. e438598.

47.	Zabora, J., et al., *The prevalence of psychological distress by cancer site.* Psychooncology, 2001. **10**(1): p. 19-28.

48.	Bauer, M.R., et al., *Quality of Life in Patients With Pancreatic Cancer and Their Caregivers: A Systematic Review.* Pancreas, 2018. **47**(4): p. 368-375.

49.	Divani, A., et al., *Effect of cancer treatment on sleep quality in cancer patients: A systematic review and meta-analysis of Pittsburgh Sleep Quality Index.* Supportive Care in Cancer, 2022. **30**(6): p. 4687-4697.

50.	Grossberg, A.J., et al., *Multidisciplinary standards of care and recent progress in pancreatic ductal adenocarcinoma.* CA Cancer J Clin, 2020. **70**(5): p. 375-403.

51.	Luo, H., et al., *Exercise Medicine in the Management of Pancreatic Cancer: A Systematic Review.* Pancreas, 2021. **50**(3): p. 280-292.

52.	Bettison, T.M., et al., *Understanding the Pathophysiology of Psychological Distress and Pancreatic Cancer: A Systematic Review.* Pancreas, 2018. **47**(4): p. 376-381.

53.	Guloksuz, S., L.K. Pries, and J. van Os, *Application of network methods for understanding mental disorders: pitfalls and promise.* Psychol Med, 2017. **47**(16): p. 2743-2752.

54.	van der Lee, M.L. and M.P.J. Schellekens, *Capturing the complexity of mental disorders in the medically ill: the network approach on behavioral medicine.* Translational Behavioral Medicine, 2019. **10**(3): p. 812-816.

55.	Hunt, M., J. Auriemma, and A.C.A. Cashaw, *Self-Report Bias and Underreporting of Depression on the BDI-II.* Journal of Personality Assessment, 2003. **80**(1): p. 26-30.

56.	HINZ, A., et al., *Anxiety and depression in cancer patients compared with the general population.* European Journal of Cancer Care, 2010. **19**(4): p. 522-529.

57.	Hinz, A., et al., *Age and gender differences in anxiety and depression in cancer patients compared with the general population.* Eur J Cancer Care (Engl), 2019. **28**(5): p. e13129.

58.	Abboud, Y., et al., *Increasing Pancreatic Cancer Incidence in Young Women in the United States: A Population-Based Time-Trend Analysis, 2001-2018.* Gastroenterology, 2023. **164**(6): p. 978-989 e6.

59.	Sung, H., et al., *Emerging cancer trends among young adults in the USA: analysis of a population-based cancer registry.* Lancet Public Health, 2019. **4**(3): p. e137-e147.

60.	The Lancet Gastroenterology, H., *Cause for concern: the rising incidence of early-onset pancreatic cancer.* Lancet Gastroenterol Hepatol, 2023. **8**(4): p. 287.

61.	Titov, N., et al., *Psychometric comparison of the PHQ-9 and BDI-II for measuring response during treatment of depression.* Cogn Behav Ther, 2011. **40**(2): p. 126-36.

62.	Skapinakis, P., *Spielberger State-Trait Anxiety Inventory*, in *Encyclopedia of Quality of Life and Well-Being Research*, A.C. Michalos, Editor. 2014, Springer Netherlands: Dordrecht. p. 6261-6264.

63.	Derogatis, L.R., *Symptom checklist-90-revised, brief symptom inventory, and BSI-18.* Handbook of psychological assessment in primary care settings, 2017: p. 599-629.

64.	Wang, X., et al., *Prognostic value of depression and anxiety on breast cancer recurrence and mortality: a systematic review and meta-analysis of 282,203 patients.* Mol Psychiatry, 2020. **25**(12): p. 3186-3197.

65.	Dinesh, A.A., et al., *Anxiety, depression and urological cancer outcomes: A systematic review.* Urol Oncol, 2021. **39**(12): p. 816-828.

66.	Trudel-Fitzgerald, C., et al., *Anxiety, Depression, and Colorectal Cancer Survival: Results from Two Prospective Cohorts.* J Clin Med, 2020. **9**(10).

67.	Kamath, S.D., S.M. Kircher, and A.B. Benson, *Comparison of Cancer Burden and Nonprofit Organization Funding Reveals Disparities in Funding Across Cancer Types.* J Natl Compr Canc Netw, 2019. **17**(7): p. 849-854.

68.	McIntosh, S.A., et al., *Global funding for cancer research between 2016 and 2020: a content analysis of public and philanthropic investments.* Lancet Oncol, 2023. **24**(6): p. 636-645.

69.	Wang, Y.-H., et al., *Depression and anxiety in relation to cancer incidence and mortality: a systematic review and meta-analysis of cohort studies.* Molecular Psychiatry, 2020. **25**(7): p. 1487-1499.

70.	Hegyi, P., et al., *Accelerating the translational medicine cycle: the Academia Europaea pilot.* Nat Med, 2021. **27**(8): p. 1317-1319.

71.	Hegyi, P., et al., *Academia Europaea Position Paper on Translational Medicine: The Cycle Model for Translating Scientific Results into Community Benefits.* J Clin Med, 2020. **9**(5).

Generally

**General Strengths:**

The study addresses an important gap in psycho-oncology literature with clear clinical relevance

Comprehensive methodology following PRISMA guidelines with PROSPERO registration

Large sample sizes across multiple psychological domains (n=78,930 for depression)

Rigorous statistical approach accounting for heterogeneity and bias

**Abstract:**

The abstract would benefit from specifying the exact databases searched and inclusion dates

Consider adding the Joanna Briggs Institute tool as your quality assessment method

The conclusion could be strengthened by mentioning specific intervention recommendations

**Introduction**:

Excellent justification of study importance with current epidemiological data

Good coverage of psycho-oncology service gaps in Europe

Consider adding a sentence explicitly stating why pancreatic cancer patients may be particularly vulnerable to psychological distress

**Methods:**

Very thorough and reproducible methods section

Minor note: "frequents" should be "frequentist" in the abstract methods

The CoCoPop framework explanation is clear and appropriate

Excellent handling of heterogeneity through random-effects models

**Results**:

The confidence interval formatting needs consistency (use either proportions or percentages, not both)

Consider adding prediction intervals where possible given the high heterogeneity

The forest plots and supplementary materials appear comprehensive

The risk of bias assessment could be highlighted more prominently in the main text

**Discussion:**

Should explicitly address the clinical implications of the high prevalence rates found

Consider adding a limitations paragraph discussing:

Potential measurement variability across studies

Possible underreporting of psychological symptoms

Generalizability given most studies from certain regions

The conclusion could be strengthened by specific policy recommendations

**Additional Suggestions**:

Consider a supplemental table summarizing measurement tools used across studies

The high heterogeneity warrants discussion of potential sources (e.g., measurement differences, cultural factors)

Consider adding GRADE assessment of evidence certainty
