# Supplementary material for: One out of four patients with pancreatic cancer experience psychological symptoms: A systematic review and meta-analysis
Source: PLoS One. 2026 May 27;21(5):e0348435. doi: 10.1371/journal.pone.0348435 (PMC13215498; doi:10.1371/journal.pone.0348435)
Supplement: Attachment — Submitted filename: Response_to_Reviewers.docx [file pone.0348435.s018.docx]

Dear Editor,

Dr Cho Lee Wong,

Thank you for giving us the opportunity to submit a revised draft of our manuscript titled *One out of four patients with pancreatic cancer experience psychological symptoms: a systematic review and meta-analysis* to *PLOS ONE*. We appreciate the time and effort that you and the reviewers have dedicated to providing your valuable feedback on our manuscript. We are grateful to the reviewers for their insightful comments on our paper. We have been able to incorporate changes to reflect most of the suggestions provided by the reviewers. We uploaded a marked-up copy of the manuscript that highlights changes made to the original version, labeled as 'Revised Manuscript with Track Changes'.

As well as, an unmarked version of the revised paper without tracked changes, labeled as ‘'Manuscript'.

Here is a point-by-point response to the reviewers’ comments and concerns.

Again, thank you for your very valuable time invested in our manuscript.

Best regards,

Péter Hegyi and his team.

Dear Reviewers,

Thank you very much for your valuable feedback. We greatly appreciated your comments which have significantly improved our manuscript. Please find below our point-by-point responses. As all comments appeared under “By Author,” we were unsure which comment belonged to which reviewer. Therefore, we have addressed the comments in the order in which they appeared.

We hope that our revised manuscript will meet your expectations and you will find it suitable for publication. Please let us know if any further changes are required.

Again, thank you for your very valuable time invested in our manuscript.

Best regards,

Péter Hegyi and his team.

- **Comment 1:** “*Many systematic reviews there about Psychological stress and pancreatic cancer patients but more interested if revies foucosed on why one patients expericed from the four patients”*

**Response 1:** Thank you for your comment. We would like to clarify that the goal of our systematic review and meta-analysis was to estimate the prevalence of psychological symptoms among patients with pancreatic cancer, rather than to identify individual-level predictors of psychological distress. The title “*One out of four patients with pancreatic cancer experience psychological symptoms*” was formulated to reflect our key findings. We agree that exploring the underlying factors that determine why certain patients experience psychological symptoms is an important question, but it was beyond the scope of this review.

**Action 1:** Not needed.

- **Comment 2:** “*Try to avoid an abbreviation from the abstract.”*

**Response 2:** Thank you for your suggestion — we fully agree. In the original abstract, we used two abbreviations: PC (pancreatic cancer), which appeared three times, and CI (confidence interval), which was used six times. We have now removed the abbreviation PC and replaced it with the full term. Given the frequent use of CI, and its widespread recognition in academic writing, we have retained it to ensure clarity and readability.

**Action 2:** We removed the abbreviation PC from the abstract and replaced it with the full term pancreatic cancer. We retained CI (confidence interval) due to its frequent use and common understanding in scientific literature. *We left the PC (pancreatic cancer) abbreviation in the text (apart from the abstract) as it appears 22 times, in order to improve the readability and flow of the manuscript.

- **Comment 3: “***Background (first Paragraph) The first sentence is factual but could better link to the study's focus on psychological burden. Try to revision”*

**Response 3:** Thank you for your valuable comment regarding the first paragraph of the abstract. We appreciate your suggestion to strengthen the link between the high mortality of pancreatic cancer and the psychological burden.

**Action 3:**Therefore, we added the following sentence to strengthen the link:

“*As the prognosis remains extremely poor, this trend brings not only a growing clinical challenge, but also a significant psychological toll for patients.*”

- **Comment 3:** *“Try to revise”*

**Response 3:** Thank you for your suggestion. To fully understand the request could you detail it further? Thank you in advance.

**Action 3:** None.

- **Comment 4:”** *I think this parts methodology parts so if you add like:*

*1. The use data extracted and exported must mandatory*

*2. Also what you search articles many of are you there is no others relevant studies from others searching database what you are (MEDLINE, Cochrane, and Embase) please accurately try add realy get those data reality 1.*

*3. Add the risk of bias was assessed using a methods test*

*4. Also the later results present by what? add”*

**Response 4:**

1. If we understand your comment correctly, you suggest to explicitly state that data was extracted and exported for analysis? If so, we have clarified in the abstract that data were extracted and exported for synthesis. We hope this improves the methodological transparency of our manuscript.
2. Could you please assure us that your comment means to ensure that the literature search was comprehensive enough and included all relevant studies? The PRISMA guidelines recommend searching in two databases, however, in order to find all the relevant studies, we conducted our search in more than two databases — MEDLINE, Embase, and Cochrane — the three biggest and mostly accepted databases in systematic reviews. We believe this approach ensures sufficient coverage and minimizes the risk of missing relevant literature.
3. and ***4.*** Thank you for your comment. We would kindly ask for clarification, as we are not entirely certain we have understood your suggestion and question correctly. If your comment refers to the need for more detail regarding the risk of bias assessment and the way results were presented, we would like to note that the journal imposes a strict word limit for the abstract. Therefore, we aimed to balance methodological transparency with conciseness. If further clarification is required in the abstract, we could add a sentence such as:

“*Risk of bias was assessed using the Joanna Briggs Institute Critical Appraisal Tool by two independent reviewers, with disagreements resolved by a third. A frequentist random-effects meta-analysis was performed, and results are presented with forest plots, heterogeneity statistics, and funnel plots for publication bias.*”

However, we believe that adding this 44-word sentence would exceed the abstract’s length unnecessarily and would require omitting other key content.

**Action 4:** *1*. The following sentence was added: “*Relevant data were extracted and exported for quantitative synthesis*.”

*2., 3., 4.*: None.

- **Comment 5:** *“Make clarify please*

*1. Double Percentage Problem: When you write "27%; CI= [0.18-0.39%]", you are First converting the proportion to 27% (correct). But then showing the CI bounds as percentages of a percentage (0.18% of 27%), which is wrong. Generally for all results write: Correct Formats (Choose One): Proportion Format (Preferred for Meta-Analyses): 0.27 (95% CI: 0.18-0.39) Most statistically accurate, Avoids percentage confusion and Common in epidemiology literature. 2. Percentage Format (If Required): 27% (95% CI: 18%-39%) More intuitive for clinicians, Must multiply decimals by 100 and Keep "%" on both estimate and CI bounds. Correct interpretation If your analysis gave a proportion of 0.27 (27%) with CI [0.18-0.39], these decimals are already proportion”*

**Response 5:** Thank you for highlighting this. We fully agree with your suggestion.

**Action 5:** We now made the correction and used the following format: *0.27 (95% CI: 0.18-0.39)*

- **Comment 6: “***Why? Combined two sentences into one cohesive thought. Removed redundant phrasing (most and least effective... beneficial approaches).????????”*

**Response 6:** We understand your suggestion to improve clarity and reduce redundancy in the final sentences of the abstract.

**Action 6:** In response, we have merged the two sentences into a more cohesive conclusion and recommendation, in line with standard expectations for systematic reviews, see next comment (**Comment 7**).

- **Comment 7: “***Make sure the contents have conclusion and recommendation format of systematic review?”*

**Response 7:** Thank you for the recommendation.

**Action 7:** We have now a better conclusion and recommendation, in line with standard expectations for systematic reviews. The revised section now reads:

“*These findings highlight the urgent need to integrate psychological care into pancreatic cancer treatment. Future research should explore sources of heterogeneity to better identify which approaches provide the most psychological benefit and tailor interventions to patient needs accordingly.*”

- **Comment 8: “***Make consistent the journals protocol point is before reference or after reference?”*

**Response 8:** Thank you for highlighting this out.

**Action 8:** We corrected the mistake accordingly.

- **Comment 9: “***Re write this protocol registration”*

**Response 9:** Thank you for your comment. We would kindly ask for clarification, as the current phrasing appears to be grammatically correct and clearly communicates both our adherence to PRISMA and the PROSPERO registration of the protocol. We would be happy to revise the sentence if you could specify what aspect of the wording you found unclear or problematic.

**Action 9:** None.

- **Comment 10: “***Use reference protocol registration on international prospective of systematic review. Also if changes like authors due to different case elaborate in this”*

**Response 10:** Thank you for your suggestion. We assume you are referring to the phrasing of the protocol registration. Our original sentence was grammatically correct and conveyed the essential information, but we are happy to revise it slightly.

**Action 10:** We included the full name of PROSPERO as “*The International Prospective Register of Systematic Reviews*” for clarity. We also confirm that no deviations (including authorship or content changes) occurred from the registered protocol.

- **Comment 11: “***My be for me weak because why not elaborate others from others journal”*

**Response 11:** Thank you for your feedback. We are not entirely sure we understood your concern correctly. If you are suggesting that our search strategy should have included more databases or sources beyond MEDLINE, Embase, and Cochrane, we would like to clarify that we deliberately focused on these three large and widely used databases in line with PRISMA recommendations, which require a minimum of two databases.

**Action 11:** None.

- **Comment 12: “***Why only focus only 3 journal for collects article 23,282? That mean there is no relevant study others journal? Example I get from Google, AJOL, Google Scholar, and Hinari and others”*

**Response 12:** We chose MEDLINE, Embase, and Cochrane CENTRAL because these are among the most comprehensive and widely used biomedical databases and are standard in systematic reviews following PRISMA guidelines. Together, they cover a broad spectrum of high-quality peer-reviewed literature, including studies from a wide range of journals and countries. We believe this approach strikes a reasonable balance between breadth and methodological quality.

**Action 12:** None.

- **Comment 13: ”***Justification why bounded between this before and afters?”*

**Response 13:** Thank you for your comment. We believe there may have been a misunderstanding: our initial systematic search was conducted on May 22, 2022, covering all available literature up to that point, regardless of publication year. Due to a delay in submission, we performed an updated search on March 3, 2025, to ensure the inclusion of the most recent studies. The search strategy was not limited to publications between these two dates — rather, the March 2025 search served to supplement the original data.

**Action 13:** None.

- **Comment 14: “***Add General characteristics of studies included in the meta-analysis”*

**Response 14:** Thank you for your comment. We would like to note that the general characteristics of the studies included in the meta-analysis are summarized in the main text and presented in detail in the Supporting Information (Table S2). The text includes the number of studies addressing each psychological domain and the range of participant numbers. Additional studies included in the systematic review are detailed in Table S3.

As these tables are self-explanatory and clearly referenced in the manuscript, we believe they provide sufficient background without unnecessarily extending the main text. However, if there are specific details you find missing or unclear, we would be grateful for further clarification.

**Action 14:** None.

- **Comment 15: “***Clearly write the quality score of the study with draws tables”*

**Response 15:** Thank you for your comment. We would like to respectfully note that the methodological quality assessment is already mentioned in the manuscript, and the table in the Supplementary information was mentioned (Table S4) too. However, we understand that it might be concise.

**Action 15:** We rewrote the section: *“The quality of the included studies was assessed using the Joanna Briggs Institute Critical Appraisal Tool. Detailed scores and assessment outcomes are provided in Supporting Information, Table S4. Studies were grouped by risk of bias (high, medium, low), and these groupings are also reflected in the forest plots.”* We believe this presentation is clear and appropriately detailed for the reader.

- **Comment 16:** *“The same above comments unders abstract of results Double Percentage Problem”*

**Response 16:** Thank you for highlighting this. We fully agree with your suggestion.

**Action 16:** We made the correction accordingly.

- **Comment 17:** *“Add sensitivity analysis. Clearly”*
- **Response 17:** Thank you for your comment. We acknowledge that the Leave-one-out analysis is a well-established sensitivity analysis method. While the manuscript reports the Leave-one-out results, we agree that explicitly labeling it as a sensitivity analysis would improve clarity.
- **Action 17:** We added the term sensitivity analysis to the heading: “*Publication Bias, Heterogeneity, and Sensitivity Analyses*”
- **Comment 18:** *“Add Author’s contribution: Abbreviation and acronyms (optional) but I see in abstract like PC, PRISMA”*
- **Response 18:** Thank you for your comment. The Author Contributions section was completed as part of the submission process on the platform. Regarding abbreviations and acronyms, we have used only a few, all of which are clearly defined in the manuscript. For example, “PC” is defined on first use. We also note that “PRISMA” does not appear in the abstract, so no abbreviation for it is included there.
- **Action 18:** None needed

**Additional clarifications**

- **Comment 19:**

***“General Strengths:***

*The study addresses an important gap in psycho-oncology literature with clear clinical relevance.*

*Comprehensive methodology following PRISMA guidelines with PROSPERO registration*

*Large sample sizes across multiple psychological domains (n=78,930 for depression)*

*Rigorous statistical approach accounting for heterogeneity and bias”*

- **Response 19:** Thank you for these encouraging comments and greatly appreciate the recognition of our study’s strengths.
- **Action 19:** Not needed.
- **Comment 20:**

***“Abstract:***

*The abstract would benefit from specifying the exact databases searched and inclusion dates*

*Consider adding the Joanna Briggs Institute tool as your quality assessment method*

*The conclusion could be strengthened by mentioning specific intervention recommendations”*

- **Response 20:** Thank you for these helpful suggestions. Due to the word count limit, we were unable to add all the suggested information and prioritized including details regarding the study’s quality assessment. Regarding the suggestion to include intervention recommendations, we respectfully note that, due to the word count limit and the lack of clearly defined, evidence-based interventions, we did not add this to the abstract. Indeed, one of the central points of our study is precisely the need for future research to identify and evaluate effective interventions for this highly burdened patient group.
- **Action 20:** We added the following sentence to the abstract: the “*Study quality was assessed using the Joanna Briggs Institute tool.”*
- **Comment 21:**

***“Introduction****:*

*Excellent justification of study importance with current epidemiological data*

*Good coverage of psycho-oncology service gaps in Europe*

*Consider adding a sentence explicitly stating why pancreatic cancer patients may be particularly vulnerable to psychological distress”*

- **Response 21:** Thank you for your comments and suggestions, we fully agree.
- **Action 21:** We have added the following sentence to the Introduction to highlight the particular vulnerability of pancreatic cancer patients: *“due to the disease’s poor prognosis, rapid progression, and high symptom burden*.”
- **Comment 22:**

**“Methods:**

Very thorough and reproducible methods section

Minor note: "frequents" should be " in frequentist” the abstract methods

The CoCoPop framework explanation is clear and appropriate

Excellent handling of heterogeneity through random-effects models”

- **Response 22:** We would like to clarify that the phrase “in frequentist random-effects models” has always been correctly stated in the manuscript, and there is no instance of “frequents.” If we have misunderstood your comment and a correction is needed, please let us know.
- **Action 22:** No need.

- **Comment 23:**

**“Results**:

1.The confidence interval formatting needs consistency (use either proportions or percentages, not both)

2.Consider adding prediction intervals where possible given the high heterogeneity

The forest plots and supplementary materials appear comprehensive

3.The risk of bias assessment could be highlighted more prominently in the main text”

- **Response 23:** Thank you for your valuable feedback.

We fully agree with the confidence interval formatting.

Regarding the prediction intervals in random-effects meta-analysis, the estimation of the prediction interval relies on the between-study heterogeneity parameter (tau-square), which represents the variance of the random effects. When the number of included studies is small (e.g., fewer than five), or when the number of studies is somewhat larger, but tau-square is substantial relative to the within-study sampling error (e.g., with fewer than 8–10 studies), the estimation of tau-square may be imprecise and unstable.  In all other cases, we reported the prediction interval (at least on the forest plot) to reveal the absolute heterogeneity.

We understand the need to highlight this information more.

- **Action 23:**

1. We now made the correction and used the following format: “*0.27 (95% CI: 0.18-0.39)”*

2. None.

*3. We added the following: “The quality of the included studies was assessed using the Joanna Briggs Institute Critical Appraisal Tool. Detailed scores and assessment outcomes are provided in Supporting Information, Table S4. Studies were grouped by risk of bias (high, medium, low), and these groupings are also reflected in the forest plots.”* We believe this presentation is clear and appropriately detailed for the reader.

- **Comment 24:**

**“Discussion:**

Should explicitly address the clinical implications of the high prevalence rates found

Consider adding a limitations paragraph discussing:

Potential measurement variability across studies

Possible underreporting of psychological symptoms

Generalizability given most studies from certain regions

The conclusion could be strengthened by specific policy recommendations”

- **Response 24:** We appreciate your suggestion to include these important points. We would like to politely point out that our manuscript already addresses the clinical implications, limitations within the '*Strengths and limitations*' section and the main body of the discussion. Regarding the potential measurement variability across studies, we have stated in our discussion:

“*As for the limitations...differences in the assessment tools and criteria used across studies can impact the prevalence. The articles' definitions of symptoms were variable and vague, measured with different tools, and severity thresholds varied.*” We also elaborate on this in the main discussion:

“*The measurement tools included in the studies are widely used in clinical and research settings... variations in administration protocols, scoring methods, cultural adaptations, and severity across different studies can further complicate the comparability and interpretability of findings from these instruments.*”

Regarding the possible underreporting of psychological symptoms, we have addressed this in the discussion by noting:

“*Psychological symptoms, such as depression, anxiety, and distress, might be underreported due to stigma or reluctance to address mental health issues. Cancer patients, including those with pancreatic cancer, may be hesitant to acknowledge or seek help for emotional challenges, which may lead to an underestimation of the prevalence of the symptoms.*”

Regarding the generalizability given most studies from certain regions, we have discussed this point in relation to heterogeneity:

“*The heterogeneity observed in the meta-analysis suggests that patient populations are diverse across studies. Differences in demographics, disease stages, treatment modalities, and geographic locations can impact reported values. A more homogeneous population can provide a more accurate value for prevalence, as cultural and societal factors influence how individuals express and perceive psychological symptoms, as seen in the studies included with high or low prevalence, e.g., in the study by Akizuki et al.[16] Variations in cultural norms, attitudes toward mental health, and the availability of support services can contribute to differences in reported proportions across studies.*”

We agree that specific policy recommendations are crucial for strengthening the conclusion. We believe that we included these recommendations in our 'Implications for practice, research and policymakers' section.

We believe these points comprehensively cover the suggested limitations. We can consider rephrasing or consolidating these points to be more explicit within a dedicated limitations paragraph if you feel is necessary.

- **Action 24: None.**
- **Comment 25:**

**“Additional Suggestions**:

Consider a supplemental table summarizing measurement tools used across studies

The high heterogeneity warrants discussion of potential sources (e.g., measurement differences, cultural factors)

Consider adding GRADE assessment of evidence certainty”

- **Response 25:**

We appreciate your suggestion to include a supplemental table summarizing the measurement tools. We would like to note that we have already included the measurement tools used in each study within our baseline characteristics section (Table S2). The information is readily available for readers who wish to examine the tools used in each individual study.

We believe we have already extensively discussed the potential sources of heterogeneity in our manuscript. In the discussion section, we have dedicated a paragraph to this topic. We feel that creating a separate table would be redundant and would not significantly add to the value of our manuscript.

Regarding the GRADE assessment of evidence certainty, we would like to respectfully point out that GRADE is primarily designed for assessing the certainty of evidence for interventions and their effects (e.g., in randomized controlled trials), where it evaluates factors like risk of bias, imprecision, and inconsistency in effect estimates. Our study is a systematic review and meta-analysis of prevalence, which is a descriptive epidemiological question. For this type of research, the methodological quality of the included studies is more appropriately assessed using tools specifically developed for this purpose. For this reason, we chose to use the Joanna Briggs Institute (JBI) Critical Appraisal Checklist for Prevalence Studies, which we believe is the most appropriate and widely recognized tool for assessing the risk of bias in prevalence studies.

**Action 25:** None

We would like to thank you once again for your time and thoughtful comments. Your feedback has been invaluable in helping us to strengthen the manuscript. We look forward to hearing from you in due time regarding our submission and to respond to any further questions and comments you may have.

Sincerely,

Péter Hegyi and his team.
