# Supplementary material for: One out of four patients with pancreatic cancer experience psychological symptoms: A systematic review and meta-analysis
Source: PLoS One. 2026 May 27;21(5):e0348435. doi: 10.1371/journal.pone.0348435 (PMC13215498; doi:10.1371/journal.pone.0348435)
Supplement: Attachment — Submitted filename: Comments to the Author(s)-PONE-D-25-25313R1.docx [file pone.0348435.s019.docx]

 Below, I outline my major concerns and suggestions for revision.

Major Comments:

1. **Statistical Presentation of Proportions and Confidence Intervals**
   Although the authors have corrected the format of confidence intervals in the abstract (e.g., from “27%; CI=[0.18–0.39%]” to “0.27 (95% CI: 0.18–0.39)”), this correction has **not been consistently applied throughout the manuscript**. For instance, in the Results section (pp. 25–27), the same error persists (e.g., “proportion was 27%; [95% CI: CI=[0.18-0.39]%”).
   - **Recommendation:** Please ensure that all prevalence estimates and confidence intervals are reported consistently in the **proportion format** (e.g., 0.27, 95% CI: 0.18–0.39) rather than mixing percentages and proportions. This is essential for statistical accuracy and reader comprehension.
2. **Clarity and Completeness of the Methods Section**
   - The authors mention the use of the **Joanna Briggs Institute (JBI) tool** for quality assessment but do not provide a summary or interpretation of the results in the main text. The reader is referred to Supplementary Table S4, but a brief summary of the overall quality of included studies (e.g., how many were low, moderate, or high risk) should be included in the main manuscript to enhance transparency.
   - The term **“frequentist random-effects models”** is used, but the rationale for choosing a frequentist over a Bayesian approach is not discussed. A brief justification would strengthen the methodology.
3. **Heterogeneity and Sensitivity Analyses**
   - The I² values are exceptionally high (e.g., 98.2% for depression), indicating substantial heterogeneity. The authors briefly mention heterogeneity in the Discussion but do not sufficiently explore its potential sources (e.g., differences in measurement tools, cultural contexts, study populations).
   - **Recommendation:** A more in-depth discussion of the possible reasons for heterogeneity is needed. Consider conducting subgroup or meta-regression analyses (if feasible) to explore sources of variation, such as geographic region, assessment tool, or time since diagnosis.
4. **Clinical and Policy Implications**
   - The authors appropriately call for integrating psychological care into pancreatic cancer treatment. However, the recommendations remain general.
   - **Recommendation:** Provide more specific, actionable suggestions for clinicians and policymakers. For example:
     - Routine screening for psychological symptoms using validated tools.
     - Referral pathways to mental health services.
     - Training for oncology care teams in basic psychological support.
5. **Limitations Section**
   - The limitations are mentioned briefly but should be expanded. Key issues include:
     - High heterogeneity among studies.
     - Potential underreporting of psychological symptoms due to stigma.
     - Overrepresentation of certain geographic regions (e.g., Europe and North America), limiting generalizability.
     - Variability in measurement tools and diagnostic thresholds.
6. **Data Availability Statement**
   - The current statement, “Data available in original articles,” is not sufficient under PLOS ONE’s data policy.
   - **Recommendation:** Please revise to state clearly that all data extracted for the meta-analysis are included in the manuscript and/or Supporting Information files. If data are only available from original studies, this must be explicitly stated with reasons.

Minor Comments:

- **Abstract:** The sentence “Study quality was assessed using the Joanna Briggs Institute tool” is appropriate but should specify that this was done for included studies.
- **Figures:** Ensure that all forest plots and figures are clearly labeled and that prediction intervals are included where appropriate.
- **References:** Check consistency in reference formatting throughout the manuscript.
