# Supplementary material for: One out of four patients with pancreatic cancer experience psychological symptoms: A systematic review and meta-analysis
Source: PLoS One. 2026 May 27;21(5):e0348435. doi: 10.1371/journal.pone.0348435 (PMC13215498; doi:10.1371/journal.pone.0348435)
Supplement: Attachment — Submitted filename: Commenbts for Authors.docx [file pone.0348435.s022.docx]

**Comments for Authors**

Dear authors, you all did a great job. The following comments may improve the current manuscript.

1. Please provide details of excluded studies as supplementary file.
2. Please provide information about grey literature.
